# Cu_2_O/SnO_2_ Heterostructures: Role of the Synthesis Procedure on PEC CO_2_ Conversion

**DOI:** 10.3390/ma16134497

**Published:** 2023-06-21

**Authors:** Maddalena Zoli, Hilmar Guzmán, Adriano Sacco, Nunzio Russo, Simelys Hernández

**Affiliations:** 1 CREST Group, Department of Applied Science and Technology (DISAT), Politecnico di Torino, 10129 Turin, Italy; maddalena.zoli@polito.it (M.Z.); hilmar.guzman@polito.it (H.G.); nunzio.russo@polito.it (N.R.); 2 Center for Sustainable Future Technologies @Polito, Istituto Italiano di Tecnologia, 10144 Turin, Italy; adriano.sacco@iit.it

**Keywords:** CO_2_ reduction and conversion, photo-electrocatalysis, Cu_2_O stabilization, characterization

## Abstract

Addressing the urgent need to mitigate increasing levels of CO_2_ in the atmosphere and combat global warming, the development of earth-abundant catalysts for selective photo-electrochemical CO_2_ conversion is a central and pressing challenge. Toward this purpose, two synthetic strategies for obtaining a Cu_2_O–SnO_2_ catalyst, namely co-precipitation and core–shell methods, were compared. The morphology and band gap energy of the synthesized materials were strongly different. The photoactivity of the core–shell catalyst was improved by 30% compared to the co-precipitation one, while its selectivity was shifted towards C_1_ products such as CO and formate. The stability of both catalysts was revealed by an easy and fast EIS analysis, indicating how the effective presence of a SnO_2_ shell could prevent the modification of the crystalline phase of the catalyst during PEC tests. Finally, directing the selectivity depending on the synthesis method used to produce the final Cu_2_O–SnO_2_ catalyst could possibly be implemented in syngas and formate transformation processes, such as hydroformylation or the Fischer–Tropsch process.

## 1. Introduction

Human activity, particularly the rising demand for fossil fuel, strongly contributes to climate change [1]. While fossil fuels have been crucial for technological, economic, and social development, their non-renewable nature and greenhouse gas emissions, particularly CO_2_, lead to global warming and its harmful effects [2,3]. There is an urgent need to reduce fossil fuel consumption and move towards green energy sources to mitigate these risks to ecosystems and humans. One promising solution to reduce CO_2_ emissions is through the conversion of CO_2_ into value-added chemicals and fuels, which can potentially contribute to a circular carbon economy. A growing research effort in electrochemical methods towards CO_2_ reduction from physical scientists and engineers is ongoing. The electrochemical pathway is considered a competitive option for CO_2_ conversion for generating valuable chemicals and fuels compared to the traditional thermochemical method [4]. This approach incorporates several important factors, including the use of mild reaction conditions, direct utilization of renewable energy sources, in situ generation of protons (H^+^) using water, and the conversion of CO_2_ into fuels or chemicals under mild reaction conditions. These strategies aim to replace conventional petroleum-derived products with more sustainable alternatives. This could result in lower infrastructure costs and the possibility of decentralized chemical production in compact, portable units. The photo-electrocatalytic (PEC) CO_2_ reduction process entails the use of a semiconductor photoelectrode to generate electrons through photoexcitation, which are then guided by an external applied potential to the electrode surface where they perform the reduction of CO_2_ [5,6]. Among the suitable semiconductors for this reaction, cuprous oxide (Cu_2_O) is a naturally p-type material with a direct band gap of 2.17 eV [7]. It possesses several advantages, including low cost, large abundance, and photo-catalytic activity under visible light irradiation [8]. As a result, it has shown potential in various applications, including photo-electrochemical and photovoltaic solar energy conversion [9]. In the last few years, it has emerged as a promising catalyst for the electrochemical reduction of CO_2_ [10]. However, a common issue observed with Cu_2_O photocathodes is the rapid decay of photocurrent within a few minutes [11,12]. This deactivation has been primarily attributed to thermodynamic factors, as the redox potentials for the reduction of Cu_2_O to Cu^0^ and the oxidation of Cu_2_O to CuO are located within the band gap [13]. In accordance with theoretical predictions, Liu and co-workers [14] observed that under illumination, Cu_2_O experiences concurrent reduction by photoelectrons and oxidation by holes within the material, leading to degradation rates that depend on the electrolyte employed. To address this issue, various strategies have been developed, such as protective over-layer coating, heterojunction engineering, crystal facet modulation, and surface modification. Broadly, scientists have aimed to provide a protection scheme to mitigate degradation. An outer shell made of a suitable material can be deposited on the Cu_2_O to form a type-II heterostructure with a staggered gap, which facilitates the transfer of photo-generated electrons from the Cu_2_O core to the outer shell, thus protecting the catalyst and enhancing its charge transfer rate [15]. Modifying oxide-derived copper with a precise amount of tin oxide has been shown to improve its Faradaic efficiency for CO production, which has been applied to 0D-Cu to produce “earth-abundant” catalysts [16,17,18]. SnO_2_ is a suitable candidate for this purpose as it is a readily available metal oxide and an intrinsic n-type semiconductor. However, achieving a uniform coverage of the core material and developing a cost-effective and scalable synthesis method remain challenging. Studies have shown that solid-state heterojunctions based on copper oxide can efficiently separate photo-generated holes and electrons. The idea behind this particle design was to create Cu_2_O particles in the form of cubic nanoparticles and then add a thin layer of SnO_2_. The SnO_2_ layer should protect the Cu_2_O particles from reduction, allowing them to perform CO_2_ reduction for longer. To this end, two methods for synthesizing the Cu_2_O–SnO_2_ catalyst are proposed in this paper.

The first one is the chemical co-precipitation (CP) technique which is employed to fabricate materials by leveraging nucleation kinetics. This methodology entails the concurrent operation of several processes, such as nucleation, growth, coarsening, and agglomeration, in a uniform solution. The size, shape, and size distribution of produced nanostructures are influenced by various factors, such as temperature, pressure, concentration, pH, stirring speed, reaction time, the nature of precursors, and the presence of surfactants or growth inhibitors [19]. These reactions usually produce insoluble products at high supersaturation conditions, and nucleation is a critical step [20]. Despite being a widely employed method due to its advantages such as simplicity, rapid preparation, easy control of particle size and composition, overall homogeneity, low temperature, energy efficiency, and the absence of organic solvents, achieving controlled nanostructures can be challenging. In fact, the co-precipitation method also has certain disadvantages, such as the possibility of trace impurities being precipitated along with the product, the time-consuming nature of the process, batch-to-batch reproducibility issues, and difficulty when reactants have different precipitation rates. To tackle these issues, for the synthesis of a photoactive Cu–Sn oxide-based catalyst made by CP for use in photo-electrocatalytic CO_2_ reduction, we decided to use the ultrasound-assisted co-precipitation method developed by Guzmán et al. [21]. A pioneering work aimed at understanding how the power of ultrasound irradiation affects the properties of a co-precipitated CuO–ZnO–Al_2_O_3_/HZSM-5 catalyst based on a similar catalyst was reported by Allahyari et al. [22]. Ultrasounds have been used for synthesizing different kinds of materials. Pinjari et al. [23] worked on nanostructured titanium dioxide using conventional and ultrasound-assisted sol-gel methods. They found that the ultrasound-assisted process has a higher yield due to improved mass transfer and the development of an ordered surface morphology. Further, Dasireddy and Likozar compared the use of co-precipitation, ultrasonic, sol-gel, and solid-state methods for the synthesis of Cu/Zn/Al catalysts for the hydrogenation of CO_2_ to methanol. They realized that the ultrasonic method resulted in improved dispersion of copper particles, higher numbers of active sites, and significant selectivity towards methanol compared to other synthesis techniques. These findings motivated the use of ultrasound-assisted co-precipitation to synthesize new catalysts for the photo-electrocatalytic conversion of CO_2_.

The second method involved in synthesizing the PEC catalyst was a core–shell (CS) approach. Core–shell nanostructures (CSNs) have traditionally been defined as composite nanomaterials consisting of cores (inner material) and shells (outer-layer material) at the nano-scale [24]. With the growing interest in task-specific nanomaterials with multifunctional capabilities and enhanced properties, CSNs are increasingly gaining attention for their versatile compositions and structures that make them suitable for these various purposes. The (photo)-electrocatalytic activity of these materials relies on the synergistic interactions between cores and shells. The specificity of this process involved the simultaneous employment of “coordinated etching”, which was first introduced by Wang et al. [25] as a low-cost method to create hollow micro/nanostructures. This method uses Cu_2_O nanocubes as a template to grow the SnO_2_ layer by dissolving the tin oxide precursor SnCl_4_ in an aqueous solution containing the Cu_2_O nanoparticles. Such solution contains CuCl, formed as an intermediate by adding the Cu_2_O NPs in a NaCl aqueous solution by coordinating Cu^+^ with Cl^−^. Zhang et al. [26] later modified the synthesis method and presented it as a versatile way to prepare catalyst materials for CO_2_ reduction. They modified a well-established method for synthesizing Cu_2_O nanoparticles by wet precipitation and adjusted the coordinated etching process to allow for the growth of tin oxide without fully dissolving the Cu_2_O particles. Cu_2_O–SnO_2_ core–shell nanocrystals were synthesized using a two-step method that involved wet precipitation followed by a modified coordinated etching process. This approach is cost-effective, straightforward, and it can be easily conducted in a laboratory beaker under atmospheric conditions, rendering it suitable for scale-up purposes. In this work, the two synthetic techniques for preparing PEC Cu–Sn-based catalysts were compared in terms of the physico-chemical characteristics and morphology of the prepared materials. This work aims to provide a reliable and comprehensive comparison of the photo-electrocatalytic properties of Cu_2_O–SnO_2_ catalysts exhibiting distinct morphologies, thereby shedding light on their significance in terms of photoactivity and charge transport and transfer properties. The unique contribution of this study lies in its in-depth exploration of these catalysts’ diverse morphologies and their direct impact on PEC performance, differentiating it from previous research endeavors in the field.

## 2. Materials and Methods

### 2.1. Reagents

The two experiments used potassium bicarbonate (KHCO_3_, 99.7%) and potassium hydroxide (KOH, 98%) as catholyte and anolyte, respectively. Nafion^®^ 117 solution (5 wt.%) and ethanol were employed to prepare the catalytic ink. The catalysts were supported on Toray carbon paper 060 for the preparation of the electrodes. Ultrapure type 1 water (MilliQ) was used to prepare all the aqueous solutions and to clean dedicated glassware and instruments. A silicone oil (purchased from Merk Life Science S.r.l., Milan, Italy) bath was used during the synthesis to keep the temperature constant.

#### 2.1.1. Co-Precipitation Method

Copper(II) nitrate trihydrate (Cu(NO_3_)_2_·3H_2_O, 99.5%), tin(IV) chloride pentahydrate (SnCl_4_⋅5H_2_O, 98%), sodium carbonate (Na_2_CO_3_, 99.5%), and sodium borohydride (NaBH_4_, 10–40 mesh, 98%) were used for catalyst preparation. All the materials were purchased from Sigma-Aldrich (Milan, Italy) and used as received unless otherwise specified.

#### 2.1.2. Core–Shell Method

Copper(II) chloride (CuCl_2_, 99%), sodium hydroxide (NaOH, ≥98%), and L-ascorbic acid (C_6_H_8_O_6_, ≥98%) were used in the Cu_2_O nanocube synthesis. To perform the second step of the synthesis, the previously obtained Cu_2_O nanocrystals were needed, in addition to ethanol (C_2_H_6_O, ≥99.8%), tin(IV) chloride pentahydrate (SnCl_4_·5H_2_O, 98%) as a tin oxide precursor, and sodium chloride (NaCl, ≥99.5%) solution. All reagents were purchased from Sigma-Aldrich (Milan, Italy) and used as received unless otherwise specified. Cellulose nitrate membrane filters (Whatman^®^ purchased from Merk Life Science S.r.l. Milan, Italy), with a pore diameter of 0.2 μm, were used for the vacuum filtration step.

### 2.2. Synthesis Procedure

Here, the method for the obtainment of the two Cu_2_O–SnO_2_ catalysts was reported. Some insights about the synthesis of the bare Cu_2_O are reported in Supporting Information (SI), Section S1.

#### 2.2.1. Co-Precipitation Method

**Figure 1:**
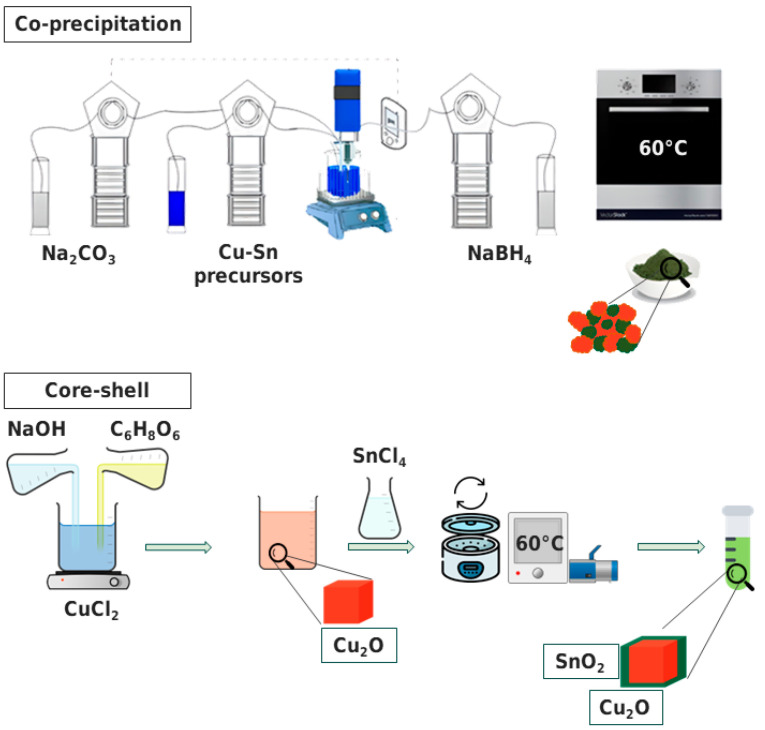
Schematic representations of the synthetic methods for the co-precipitation sample (upper part of the Figure) and for the core–shell one (lower part), together with a schematic image of each catalyst as synthesized.

Our research group previously established the methodology, and a more comprehensive description can be found in Zoli et al. [27]. The upper portion of Figure 1 presents a schematic representation of the method employed. The synthesis process involved adding a solution of 0.6 M Cu(NO_3_)_2_·3H_2_O and 0.3 M SnCl_4_⋅5H_2_O as the Cu and Sn precursors to MilliQ water heated to 70 °C, which was delivered automatically via a peristaltic pump. The solution was continuously agitated while Na_2_CO_3_ (1 M) was used as the precipitating agent. NaBH_4_ (1.2 M) was the reducing agent used to promote Cu_2_O formation. An ultrasonic processor (frequency: 20 kHz, amplitude: 30%) was utilized during the precipitation stage. The catalyst was collected through in-vacuum filtration and then it was dried at 60 °C in air. These samples were named CP_CuSn.

#### 2.2.2. Core–Shell Method

A schematic representation of the method is shown in the lower part of Figure 1. To synthesize Cu_2_O particles, a 0.02 M aqueous copper(II) chloride solution was mixed with a 6 M NaOH solution dropwise to precipitate Cu(OH)_2_, causing a rapid change from light blue to a dark blue color. After stirring for 30 min, 1.8 M ascorbic acid solution was added as a reducing agent, forming orange Cu_2_O particles. The reactor was left to age for 3 h under continuous stirring. The particles were collected by vacuum filtration and dried at 60 °C for 12 h in a Büchi in-vacuum oven. To synthesize Cu_2_O–SnO_2_ core–shell nanocrystals, the Cu_2_O particles were dispersed in 150 mL ethanol solution; then a 0.2 M NaCl solution was added to favor ion exchange and mixed with 50 mL 0.3 M tin oxide precursor (SnCl_4_·5H_2_O) solution in ethanol. The synthesized particles were separated from the solution by centrifugation and vacuum drying, resulting in a bright orange powder. Both synthesis steps were conducted in laboratory beakers under atmospheric conditions, making it feasible for scaling up. These samples were named CS_CuSn.

### 2.3. Characterization

The crystalline phase of the materials was investigated by X-ray diffraction (XRD) on a Panalytical PW 3040 X’Pert equipped with a Cu anode for K_α_ radiation generation at 40 kV operating voltage in the 2θ range of 10–100° with a scanning step of 0.013° and a Cu radiation wavelength K_α_ = 1.54018 Å. A ZEISS Supra 40 FESEM field emission scanning electron microscope (Oberkochen, Germany), equipped with an energy-dispersive X-ray spectroscopy system (EDS) operated at 3 kV, was used to obtain the morphological characterization and the contents of the relative elements of the samples. The samples were prepared by placing the catalyst powders onto a sample holder using conductive adhesive carbon tape. The molar ratios of copper and tin in the synthesized catalysts were determined by using inductively coupled plasma–optical emission spectrometry (ICP–OES). A ThermoFisher system (ICAP 7000, Thermo Fisher Scientific GmbH, Dreieich, Germany) and QTEGRA ISDS software 2.10 version were utilized. Chemical attack was carried out on a heating plate in a chemical hood by solubilizing approximately 100 mg of each sample in a mixture of 60% HCl, 20% HNO_3_, and 20% HF, followed by stirring at 100 °C for 1 h. After cooling the solution, it was diluted with MilliQ water to achieve a concentration of 1–5 ppm of each analyte. The samples were then analyzed using the instrument, and a calibration line was prepared for each analyte. Standard samples were run into the instrument for analysis. A Varian Cary 5000 spectrophotometer was used in the diffuse reflectance mode with an integrating sphere to obtain UV–Vis absorption spectra of the samples. The Tauc relation [28], reported in Equation (1), was chosen to calculate the optical band gap energy.

(1) $$\alpha h\nu =A\;{\left(h\nu -E_{g}\right)}^{n}$$

where *α* is the absorption coefficient (dimensionless unit), *hν* is the photon energy in eV, *A* is a constant related to the mass of electrons and holes, *E_g_* is the band gap energy, measured in electron Volt (eV), and the value of *n* depends on the type of transition (*n* = 1/2 for allowed direct transition and 2 for allowed indirect transition). The main textural characteristics of the materials were determined by N_2_ adsorption analysis, which included specific surface area, total pore volume, and pore size distribution. The N_2_ adsorption/desorption isotherms were measured at 77 K using TriStar II 3020 volumetric equipment (supplied by Micromeritics Instrument Corporation, Norcross, GA, USA). Before the measurement, all samples were outgassed at 200 °C for 2 h. The BET equation was used to calculate the surface area from the N_2_ adsorption/desorption isotherms. Additionally, the pore size distribution was determined using the Barrett–Joyner–Halenda (BJH) method based on the Kelvin model of pore filling.

### 2.4. Photo-Electrode Preparation

In Section S2 of Supporting Information, the results of the blank test conducted on the carbon paper support to assess its influence on the experimental outcomes are reported. The preparation of photo-electrodes involved the creation of a catalytic ink, which was then deposited onto a porous carbon support (Toray carbon paper 060) via airbrushing [29]. The ink was composed of the Cu_2_O–SnO_2_ catalyst; Nafion was used as the binder for the particles and ethanol as a dispersant. The mixture was sonicated for 30 min to achieve a uniform slurry. Each electrode had a catalyst loading of 1 mg cm^−2^ in 1 cm^2^ of the active area, which was fully illuminated with simulated sunlight during testing.

### 2.5. PEC Set-Up and Product Analysis

The experimental set-up used for the photo-electrocatalytic CO_2_ reduction test is shown in Figure S5. The scheme of the cell is also reported in the inset of Figure S5. It is a double-chamber cell (“H-type”) made in quartz illuminated from the cathode side with a simulated AM 1.5G solar light using a 450 W Xe lamp by Newport (provided by Laser-Optronic S.p.A., Milan, Italy), equipped with an AM 1.5 filter and a model 6123NS water filter, at a power density of 100 mW cm^−2^. The cell was maintained at a fixed distance from the lamp to ensure the reproducibility of tests. Gaseous CO_2_ was flowed into the cell at a flow rate of 20 NmL/min, controlled by a mass flow controller (Bronkhorst, The Netherlands), and the exit from the cell was connected to a μGC for online gas analysis. Photo-electrocatalytic CO_2_ reduction experiments were conducted in a three-electrode configuration, including the prepared photocathode as the working electrode (WE), a Pt-mesh counter electrode (CE), and an Ag/AgCl (3 M KCl) reference electrode (RE). A multichannel potentiostat (VoltaLab PGZ 301 Dynamic Potentiostat, from Radiometer Analytical SAS, Villeurbanne, FR) was used for the photo/electrochemical tests. The cell was filled in with two different electrolyte solutions: 0.1 M KHCO_3_ in water as the catholyte, kept under stirring throughout the test, and 0.1 M KOH as the anolyte. The two compartments were kept separated by a previously activated bipolar membrane provided by Fumasep. During the measurements, a constant CO_2_ flow rate was maintained to saturate the catholyte and to bring the gaseous products to the µGC (Varian 490 Micro GC, from Agilent S.p.A, Leini, TO, Italy) equipped with a PPQ and Molsieve columns). Liquid products were analyzed using a high-performance liquid chromatograph (Shimadzu HPLC, Prominence model with detector RID-10A, SPD-M20A, ELSD-LT II and RF-20A by Shimadzu Europa GmbH, Duisburg, Germany) with photodiode array detection (PDA) set at 208 nm using a Rezex ROA (300 × 7.8 mm) column, with 5 mM H_2_SO_4_ (flow rate of 0.7 mL min^−1^) as mobile phase and a gas chromatograph (Perkin Elmer GC, model Clarus 580, from Perkin Elmer, Milan, Italy) equipped with a mass spectrometer (model Clarus SQ8 S) and head space (Turbomatrix 16) by PerkinElmer Italia Spa, Milan, Italy. The Faradaic efficiency for each product was calculated by using Equation (2):

(2) $$FE\;\left(\%\right)=\frac{\left(z\;\cdot n\;\cdot F\right)}{\left(j\;\cdot A\cdot t\right)}\times 100\;$$

where *z* represents the number of electrons exchanged at the cathode surface, *n* is the outlet molar flow rate of each product, *j* is the current density (mA cm^−2^), *t* is the reaction time (s), *F* is the Faraday constant (C·mol^−1^), and *A* is the active geometric area (cm^2^). Electrochemical impedance spectroscopy (EIS) measurements were fitted through the equivalent electrical circuit reported in Section 3.2.2. The model comprises a resistance R1, representing electrolyte resistance, and a parallel combination of resistance R2 and constant phase element Q2, which characterize transport properties within the catalyst. In addition, the model also included another parallel R3//Q3 that accounts for reduction reactions [30]. The potentiostat (PalmSense4) applies a voltage signal and measures the current to evaluate the system impedance. Experiments were performed at various potentials from −0.8 to −1.5 V vs. Ag/AgCl with an AC signal of 10 mV of amplitude and its frequency was swept from 10^6^ to 10^−1^ Hz in a CO_2_-saturated 0.1 M KHCO_3_ aqueous solution under simulated sunlight illumination as for the PEC tests. The measurement campaign was repeated twice, before and after a 10 min long chronoamperometry test, carried out at the same applied potential. This assessed any changes in the impedance of the samples and to determine if there were any modifications in the catalyst during the evolution of the photo-electrocatalytic test. The results are labelled as “post” when they refer to the second part of the measurement.

## 3. Results

In this section, the primary objectives are to evaluate the two different Cu–Sn-based catalytic samples, determine of the actual quantity of Sn (%) present in the samples, confirm the dispersion of SnO_2_ onto or next to the Cu_2_O nanostructures, and identify the crystalline phases comprising the samples. Then, insights on the morphology of the two samples are provided, together with some information extrapolated from N_2_ physisorption analysis. Finally, Tauc plots give details necessary for band gap determination.

### 3.1. Physico-Chemical Comparison

**Figure 2:**
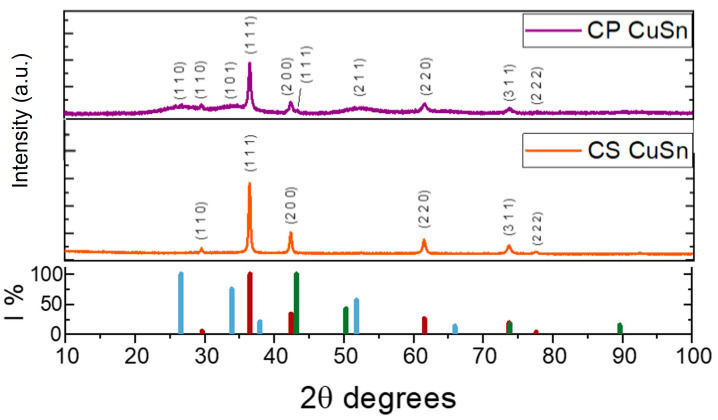
XRD of co-precipitation (CP) and core–shell (CS) samples; from the top: CP (purple line) and CS (orange line). The graph in the lower part of the figure reported the relative intensities of the Cu_2_O, Cu, and SnO_2_ peaks (red, green, and blue lines, respectively).

The catalysts were characterized by their physico-chemical properties as synthesized. The XRD pattern of the CP_CuSn sample is displayed in Figure 2 (purple line). A cubic crystalline phase (cuprite) of Cu_2_O (JCPDS 01-077-0199) was observed, with the most intense peak of the (111) plane at a 2θ value of 36.5°. No peaks associated with CuO were detected in the diffraction pattern. Additionally, a small quantity of metallic copper was present, indicated by the (111) peak at 43.3°, appearing as a shoulder of the Cu_2_O (200) peak at 42.3° (JCPDS 01-070-3039). The presence of SnO_2_ (JCPDS 00-041-1445) was also confirmed, as evidenced by its three most intense peaks at 2θ values around 26.6° (110), 33.9° (200), and 51.8° (211). The occurrence of broad peaks with a distributed bump shape, rather than narrow and highly intense peaks, could be interpreted in two ways: (1) the presence of tin oxide in its amorphous phase in the sample, or (2) the existence of very small-sized crystallites that exhibit wide diffraction peaks due to a limited number of reflection planes. Distinctive diffraction peaks observed in the XRD plot confirmed the coexistence of crystalline Cu_2_O and SnO_2_ phases, indicating a composite structure of physically mixed but distinguishable crystalline phases rather than a solid solution. The XRD spectrum of the CS_CuSn sample (orange line in Figure 2) displayed different behavior than the one observed in the co-precipitation sample. In this case, it was not possible to detect the peaks related to SnO_2_, not even those that were wide and largely distributed as in the co-precipitation sample’s diffractogram. The CS sample had the main Cu_2_O peaks, related to the (111), (200), and (220) planes at 2θ of 36.5, 42.3, and 61.5 degrees, respectively. No evidence of SnO_2_ was found. The molar ratios of bulk copper and tin in the as-synthesized catalysts were determined using ICP–OES to ascertain the presence of SnO_2_. The results of the Sn atomic percentage and the molar ratio between Cu and Sn are reported in Table 1 for both catalysts. The similarity between the two values of the Cu:Sn ratio, which was found to be equal to 2 for sample CP and equal to 3 for sample CS, justified the comparison between these two catalysts. The confirmation of the presence of Sn in the CS sample also makes the synthesis method described in the previous paragraph applicable. This confirms the hypothesis that SnO_2_ is amorphous and, therefore, not detectable by XRD. Traces of Cl were also found via ICP–OES and are present in the metallic precursors of both synthesis methods. However, when comparing the amount of Cl to those of the two main metals present, it did not exceed 2% in atomic percentage.

**Figure 3:**
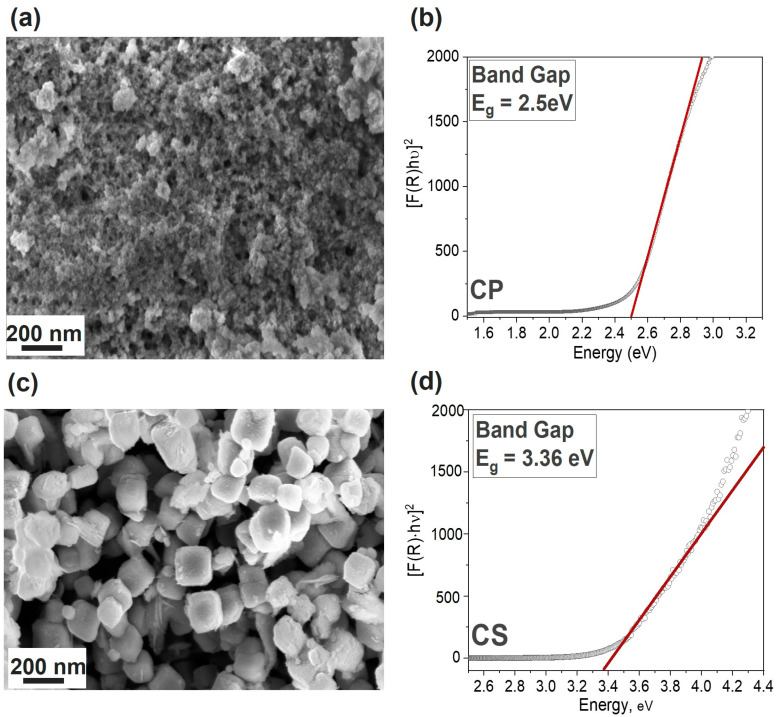
Morphology images obtained by FESEM analyses ((**a**,**c**) for CP and CS samples, respectively) and Tauc plot ((**b**,**d**) for CP and CS samples, respectively) for band gap determination In Tauc’s plots, the black dots represent the [F(R)hν]^2^ trend for each sample, while the red line is the tangent to the first linear section of the curve.

From the analysis of N_2_ adsorption/desorption isotherms, information on the specific surface area and porosity of the materials was obtained. The results for both samples are reported in Table 1. In Section S4 of Supporting Information, some insights on the BET isotherms of both catalysts are reported, together with plots of the curves (Figure S6). The BET surface area value was calculated to be 142 m^2^ g^−1^ for the CP sample which was more than double that of the CS one (64 m^2^ g^−1^). The pore volume was more similar between the two samples: 0.12 and 0.08 cm^3^ g^−1^ for CP and the CS samples, respectively. No differences were reported in the average pore width, which was 5.9 nm for both materials, as determined by the BJH method. The higher surface area and pore volume of the co-precipitation compound suggests that it could be more advantageous for the photo-catalytic activity of the material compared to the CS sample. This feature can also be observed by examining the FESEM micrographs shown in Figure 3a,c, corresponding to the catalysts obtained via co-precipitation and core–shell syntheses, respectively. In fact, in the former image, it can be observed that the catalyst exhibited a conglomeration of compact structures that represent the bulk material, accompanied by surface decoration resulting from smaller particles (with dimensions of approximately 20 nm). Within this unique structure, it was possible to identify cuprous oxide as the main component in the bulk section of the material. In contrast, the smaller particles were attributed to tin oxide. The presence of clusters of Cu_2_O and SnO_2_ on the CP_CuSn catalyst indicated again a composite structure rather than a solid solution. In Figure 3c, the core–shell catalyst micrograph is shown. With respect to the bare Cu_2_O (reported in SI, Section S1.2.2, Figure S3), an increase in the thickness of the cubes could be observed. In particular, the side of the bare nanocubes was detected to be around 90–100 nm, while in Figure 3c, the average dimension of the covered nanocubes was around 250 nm. As a result of the SnO_2_ addition step, the size of the catalyst increased by more than two times its original dimension. Moreover, the disappearance of the smoothness from the nanocube precursors is determined, and the formation of a greater amount of roughness and a generally less ordered coverage of the material are visible. This is consistent with the hypothesis that the tin oxide surface layer is amorphous, as suggested by its absence from XRD pattern. Furthermore, by comparing the results of the energy-dispersive X-ray spectroscopy (EDX) analysis on the bare CS_Cu_2_O (as reported in the Supporting Information, Section S1.2.2) with the same analysis performed on the Cu–Sn-based core–shell catalyst, it was observed that the former, without undergoing the second synthesis step, showed no traces of Sn. Instead, by analyzing the latter, a decrease in the atomic percentage of Cu from 66% to approximately 38% was observed, along with the detection of Sn at 12%. This observation, in line with the ICP–OES result reported in Table 1, confirms the structural hypothesis of a core–shell catalyst. Moreover, the optical band gap (*E_g_*) of the samples was calculated by using the Tauc’s method [31] from the F(R) spectra obtained by using a spectrophotometer in the diffuse reflectance mode with an integrating sphere. The resulting plots can be observed in Figure 3b,d for the CP and the CS catalyst, respectively. The co-precipitation catalyst presented a determined band gap value of 2.5 eV. The value was consistent with findings reported in the literature for similar structures [32]. We observed a slight energy increase with respect to the bare Cu_2_O (~2.17 eV [33]), which may be attributed to the presence of SnO_2_ on the catalyst contributing to an increase in its band gap value, owing to the higher band gap values of SnO_2_ (3.58–3.45 eV) [34]. This may serve as a beneficial characteristic to mitigate photo-corrosion issues during PEC CO_2_ reduction tests arising from the redox reaction of Cu_2_O itself [35,36]. Successfully establishing a protective layer on the surface of Cu_2_O is deemed crucial for achieving long-term catalyst durability. Regarding the core–shell catalyst (Figure 3d), the band gap energy determined via Tauc’s method amounted to 3.36 eV, about 1 eV higher compared to the bare nanocubes (2.43 eV as reported in the Tauc plot of bare CS_Cu_2_O, Supporting Information, Section S1.2.2, Figure S3). In this case, the influence of SnO_2_ on Cu_2_O was predominant. Although the catalyst evolved towards a structure with a higher band gap, characterized by decreasing ability to absorb solar radiation, this change in synthesis strategy and catalyst structure was employed to ensure a greater protection of Cu_2_O by ensuring full coverage of the Cu^+1^ species. In principle, longer stability should allow us to maintain the photoactivity, even during longer tests.

### 3.2. PEC Measurements

In this section, the results of the tests performed on both the Cu_2_O–SnO_2_ catalysts are reported and commented upon. The test protocol was designed to characterize the photoactivity of the different catalysts, aiming at maintaining their stability over time and avoiding damage due to excessive applied currents or potentials.

#### 3.2.1. PEC CO_2_ Reduction Test

**Figure 4:**
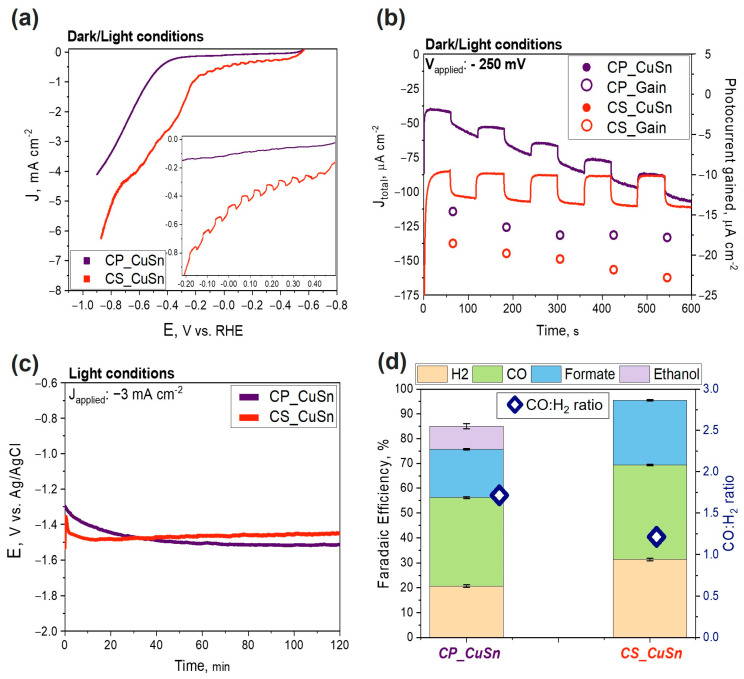
Comparison of co-precipitation (CP) and core–shell (CS) Cu_2_O–SnO_2_ photo-catalyst results; in particular: (**a**) LSV curves, (**b**) CA curves, (**c**) CP curves, and (**d**) FE of gaseous and liquid products detected after chronopotentiometry (2 h, −3 mA cm^−2^); each compound was reported together with its related uncertainty, and the CO:H_2_ ratio was reported (blue rhombus symbol, right Y axis) for both the catalysts.

In this test, the focus was on the photoactivity of the two materials, firstly evaluated via linear sweep voltammetry (LSV) (Figure 4a) and chronoamperometry (CA) tests (Figure 4b). The same aspects were evaluated by comparing the Cu–Sn-based catalysts with their relative bare Cu_2_O particles. The results of the PEC tests onto the bare Cu_2_O catalysts are reported in the Supporting Information, Section S5.1. The potential range for the LSV test was set from 0 to −0.9 V vs. RHE, the scan rate was set at 10 mV s^−1^, and the electrolyte solution was first saturated with CO_2_ for around 30 min. The solar simulator was pointed at the photo-electrode, and light/dark conditions were alternated every 2 s. Identical LSV tests were conducted in a N_2_ atmosphere. A comparison of the obtained curves for each sample in both CO_2_ and N_2_ environments can be found in Section S5.2 of the Supporting Information. Comparing the performances of both catalysts at a *J* value of −0.5 mA cm^−2^, the co-precipitation sample (purple line) presented an onset potential higher than the core–shell sample (orange line) (–0.4 V vs. 0 V vs. RHE, respectively), as shown in Figure 4a. Thus, the CS sample enabled CO_2_ reduction at lower overpotentials compared to the CP sample. Consequently, the registered current density values were higher overall (in absolute value) for the CS sample. A *J* value of around −4 mA cm^−2^ was reached in the CP sample at −0.9 V vs. RHE, while −6.5 mA cm^−2^ was observed in the CS sample by applying the same potential. The results were in line with previous ones obtained by de Brito et al. [37], where a Cu/Cu_2_O mixed film reached comparable current density values during a LSV test in 0.1 M sodium sulfate electrolyte under illumination. In agreement with the literature, a controlled-potential electrolysis carried out in the dark resulted in negligible CO_2_ reduction [38]. The LSV curves showed a typical wavy pattern (increase/decrease) due to the dark/light alternation. It can be observed in the inset of Figure 4a that the CS sample exhibited more defined steps due to a bigger gain in photocurrent. Moreover, even after the onset potential, the CS sample showed a wavy pattern, differentiating it from the CP sample, for which the increase in dark Faradaic current exceeded the photoactivity gain. CA tests were performed to determine the stability and the effective photocurrent contribution. Catalyst performances were examined with a 10 min long test, as shown in Figure 4b. The CA tests were set at an applied potential of −0.25 V vs. Ag/AgCl. The colored dots, purple and orange, standing for CP and CS, respectively, refer to the right Y axis, showing the behavior of the photocurrent gain over time. The CP catalyst presented the same photocurrent gain value as that obtained in a previous work on PEC tests onto the same catalyst [27], around 18 µA cm^−2^. The photocurrent gain was reasonably stable. The increasing current density recorded during the ten-minute test for the CP catalyst suggests that Cu(I) was gradually reduced to its metallic form Cu(0), increasing its conductivity. However, the stability of the photocurrent, measured by the difference between the current under light and dark conditions, did not change significantly during the test, indicating that the photo-catalytic activity of the catalyst was not irreversibly compromised. The increase in the total current density over time was a key difference between the two catalysts. Although the CS sample showed a slightly larger gain in photocurrent, its current density remained stable at approximately −100 µA cm^−2^. Indeed, the core–shell sample showed a photocurrent gain passing from −18 to −24 µA cm^−2^ during the duration of the test, suggesting that solar radiation may have activated the material and made it more efficient over prolonged testing periods. This could indicate the ability of core–shell synthesis to provide a more stable and functional SnO_2_ protection layer compared to the co-precipitation method. The diminished photoactivity of the CP sample can be attributed to the presence of Cu, as evidenced by the XRD pattern. The Cu species present in the CP sample hinders its photoactivity and potentially limits the increase in photocurrent during the switching on/off of the solar simulated light. It must be noted that the values are generally lower than some remarkable values found in the literature [26,39]. However, in those cases, the electrode supports were different (e.g., Cu foam or fluorine-doped tin oxide (FTO)). An agreement was noticed with the results obtained by Liu et al. [14] in their investigation on the photo-corrosion of a bare Cu_2_O catalyst in aqueous electrolytes. They performed chronoamperometry at an applied potential of −0.4 vs. RHE, achieving a photocurrent gain of around 0.1 mA cm^−2^ for the bare Cu_2_O on FTO and around 0.05 mA cm^−2^ for the Cu_2_O/FTO with Ag nanoparticles electrodeposited. Chronopotentiometry tests were also performed, and the results are reported in Figure 4c. The decision to apply a current density value of −3 mA cm^−2^ was taken based on the results of the evolution of gaseous products and the comparison of dark/light currents obtained in a previous work [27]. The achieved potential ranged from −1.5 V to −1.35 V vs. Ag/AgCl consistently, exhibiting higher values (in absolute terms) for the CP sample compared to the CS sample. The plot shows that the CP curve evolved slowly during the first 40 min and remained relatively stable during the rest of the test, while the CS sample had a sharper change in potential within the first 15 min and then remained stable at an average value of around −1.35 V vs. Ag/AgCl. Interestingly, a similar behavior was also reported by de Brito and co-workers [37]. In their work, they performed a potentiostatic measurement to evaluate the photo-electrocatalytic reduction of CO_2_ by a Cu/Cu_2_O electrode. In line with their study, the reductive photocurrent increased in the first minutes of co-electrolysis and decreased after that, reaching lower (but stable in our case) values after 120 min. This was explained by assuming the formation of intermediate CO_2_^•−^ at the beginning of co-electrolysis followed by its further reduction to generate C-compound derivatives.

To sum up, by analyzing the materials developed in the aforementioned works in comparison with that reported herein, from a photoactivity perspective, it can be concluded that a performance improvement is achieved with a rational synthesis design, moving from co-precipitation to a core–shell route. In this latter case, the stability of the material over time increased due to the creation of a proper outer protective shell. This capability, interestingly, made the catalyst a candidate for further technological developments. Additionally, the overall current density values reached (dark + light) were higher (in absolute value) for the CS catalyst, confirming a boost in efficiency compared to the CP sample. Figure 4d exhibits the Faradaic efficiencies (FEs) achieved for the tests carried out under light conditions for both catalysts. The values were obtained by applying Equation (2) after a chronopotentiometry test of 2 h at −3 mA cm^−2^. The decision to apply a current density value of −3 mA cm^−2^ was made based on the results of gaseous product evolution and of the comparison of dark/light currents obtained in preliminary tests by Zoli et al. [27]. In the work of Zoli and co-workers, it was observed that a similar current density value allowed maximizing the FE_CO_ with respect to lower or much higher values of applied *J*. Simulated sunlight acts as an additional energy source, enabling the generation and separation of photocarriers for the CO_2_ reduction reaction. It reduces the external energy consumption and facilitates the production of C1 compounds [40]. Visible light is particularly effective in exciting the CO_2_ radical anion, the initial intermediate species in CO_2_ reduction, leading to the formation of formate, CO, or C_2+_ products through a multi-step mechanism [41]. The first noticeable difference was that the total Faradaic efficiency was higher in the test conducted with the CS catalyst (rightmost part of the plot) than that conducted with the CP catalyst (around 95 vs. 85%). This could be ascribed to the fact that the core–shell catalyst presents a high intrinsic stability, likely due to the protective layer of SnO_2_. In contrast, in the test related to the CP compound, a Faradaic efficiency lower than 100% could be attributed to the reduction of Cu_2_O to Cu(0), as detected by Zoli and co-workers [27] from ex situ XRD and XPS characterizations performed on the tested electrodes to investigate the eventual modification of the copper oxidation state that occurred under PEC CO_2_R conditions. Nevertheless, this hypothesis requires further investigation and confirmation by in operando characterizations. The FE_H2_ was 1.5 times higher in the CS test than in the CP one, passing from approx. 31 to 21%, respectively. Regarding C1 products such as CO and formate, higher percentage values—albeit with a less pronounced difference—were obtained in the test conducted with the CS catalyst CS compared to that conducted with the CP sample. Specifically, in the former, the Faradaic efficiency towards CO was approximately 37%, while for formate, it was around 26%. In the latter, CO and formate accounted for approximately 35.5% and 20%, respectively. Focusing on syngas production (blue symbols linked to the right Y axis in Figure 4d), the CS sample presented a remarkable CO:H_2_ ratio~1.2, which is quite interesting regarding the industrial utilization of syngas in hydroformylation processes [42]. The CP catalyst was determined to be the most suitable candidate for further employment of methanol synthesis and Fisher–Tropsch processes because of its remarkable CO:H_2_ ratio result~1.7. Another fundamental difference between the two experiments was the presence of ethanol in the CP sample, with a Faradaic efficiency of approximately 9%. In contrast, no C_2_ products were detected in the CS sample. The presence of Cu species in the CP sample suggested that it could exhibit enhanced selectivity towards ethanol due to the catalytic properties of metallic copper and the ability of Cu/Cu^+1^ interfaces to promote *CO and *CHx intermediate dimerization towards ethanol formation in ethanol-related reactions [43]. In contrast, no C2 products were detected in the CS sample. The presence of Cu species in the CP samples, as shown by the Cu (111) peak in the diffractogram (Figure 2), suggested that it could exhibit enhanced selectivity towards ethanol due to the catalytic properties of metallic copper in ethanol-related reactions [43]. This behavior can be explained by the more homogeneous SnO_2_ shell on individual nanocube particles of cuprous oxide in the CS sample. This SnO_2_ shell not only enhances the stability of Cu^+1^ but also provides an optimal environment for catalytic CO_2_ reduction reactions, strongly directing the selectivity towards formate and C_1_ products while minimizing undesired side reactions. The synergistic effect between the core and the shell in the CS sample plays a vital role in addressing selectivity, showcasing the profound influence of morphology on catalytic performance. This finding is also supported by the work of Pardo Pérez et al. [44] who discovered that localized electronic effects at low Sn content result in the formation of Sn^δ+^, weakening CO adsorption on Cu and facilitating CO formation. For higher Sn atomic % on the surface, the overall system exhibits reactivity similar to that of pure Sn, hindering H adsorption and promoting formate production. It is clear from their study that the specific selectivity of Sn and Sn oxides addresses C_1_ products such as CO and formate more than ethanol and other C_2_ products.

#### 3.2.2. Electrochemical Impedance Spectroscopy Measurements

**Figure 5:**
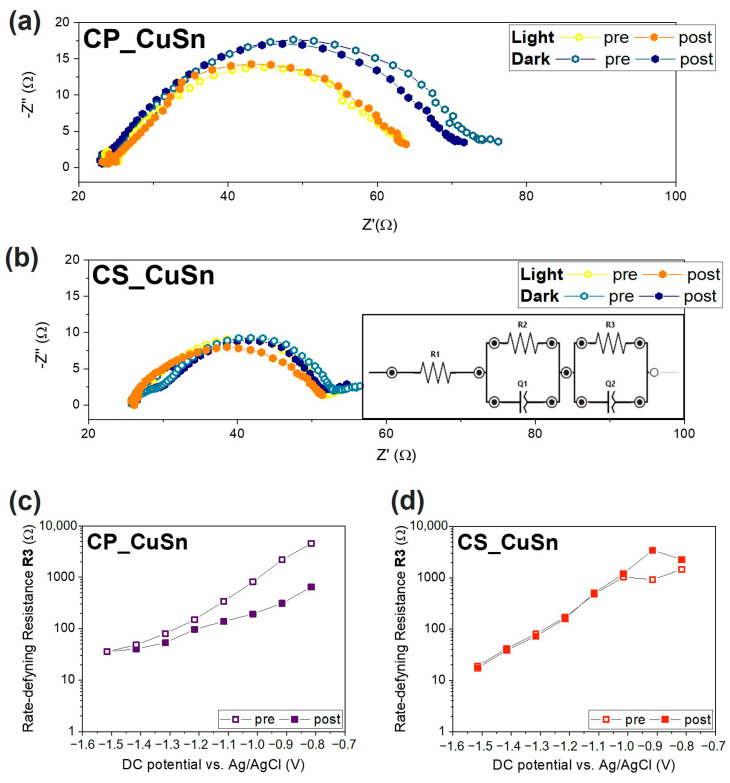
EIS analysis of the CP and CS catalysts: (**a**,**b**) Nyquist plot of CP and CS catalysts, respectively, obtained during the “pre” and “post” campaigns of measurement carried out at −1.5 V vs. Ag/AgCl in CO_2_-saturated environment both in dark and light conditions; (**c**,**d**) Equivalent resistance R3 trends of (**c**) CP and (**d**) CP samples, where open symbols represent the “pre” measurements while solid ones, labelled “post”, represent measurements executed after chronoamperometry. R3 values were calculated through a fitting procedure using the equivalent electrical circuit shown in (**b**).

Electrochemical impedance spectroscopy (EIS) in this context focused on analyzing the impedance of the samples, providing valuable insights into the stability, efficiency, and overall performance of the photo-electrocatalytic system. Changes in the EIS spectra were monitored under different operating conditions, including tests conducted both in dark and light environments, allowing for the observation of potential variations in the catalysts’ activity, degradation, or the occurrence of side reactions. This assessment helped us to understand the catalysts’ behavior and the influence of light exposure on their photo-electrochemical characteristics. In particular, for each analysis, electrochemical impedance was recorded a spectrum before (labelled as “pre”) and one after (labelled as “post”) a CA conducted at a specific applied potential, in predetermined light or dark conditions, to understand how a test could affect the stability of the catalyst. Nyquist plots measured at different potentials in the CO_2_-saturated electrolyte during simulated solar illumination are reported in the Supporting Information, Section S6, Figure S9. The plots reported in Figure 5a,b for the CP and CS catalysts show the Nyquist plots registered at −1.5 V vs. Ag/AgCl applied potential before and after a CA at the same applied potential, both in dark and light conditions. The results of the tests on the CP catalyst, Figure 5a, showed that the resistance in dark conditions was higher, with a trend towards a decreasing impedance value from “pre” to “post” measurement. In fact, the real part of impedance recorded in the “pre” test was 53 Ω, while the one detected during the “post” EIS was equal to 47 Ω. Both tests performed during the catalysts’ illumination presented a lower real impedance value, equal to 40 Ω, meaning that the whole CO_2_ reduction process was favorable when the system was exposed to solar radiation. Moreover, it was possible to state that the absence of an ordered and proper external shell, as in the CP sample, to protect the Cu^+^ phase in the Cu_2_O catalyst was deemed responsible for changes in phase during the CO_2_R tests, with Cu_2_O gradually being transformed into its metallic form. Such a hypothesis would explain the lower real impedance value due to the intrinsic high conductivity of metallic Cu and its activity in electrocatalytically reducing CO_2_. As further proof of such insight, the CS sample revealed a very low real impedance difference between “pre” and “post” measurements (see Figure 5b). The value’s variation in both tests carried out in the dark condition was lower than 1 Ω and smaller than those observed for the CP catalyst. This behavior was also observed in the light-influenced test conducted on the CS catalyst. Indeed, the real impedance values of the “pre” and “post” tests were substantially equal (~25 Ω). Again, solar radiation had a positive influence on the stability of each catalyst. The orange and yellow points shown in the graphs depict a situation of substantial similarity between the “pre” and the “post” chronoamperometry test at −1.5 V vs. Ag/AgCl. This was interpreted as confirmation that light induced a higher internal photovoltage within the catalyst, which was not used to reduce the Cu_2_O to the corresponding metallic species. By using these EIS tests as a “state-of-health” check of the catalyst during the PEC test, it can be stated that the light tests imply a lower level of modification of the catalyst and its oxidation state, and such a result is further confirmed when a proper SnO_2_ shell acting as external protection is introduced. EIS data were analyzed to gain insight into the observed behavior, fitting it to an equivalent circuit model as depicted in the inset in Figure 5b. The trends of equivalent resistance R3, calculated for all the samples, are depicted in Figure 5c,d for CP and the CS samples, respectively. Again, two lines are present in the plots, indicating those EIS tests performed before (“pre”) and after (“post”) the CA tests set at a specific value of applied potential (ranging from −0.8 to −1.5 V vs. Ag/AgCl). The empty squared symbols represent the resistance values obtained during the “pre” measurement, while the filled symbols represent the values obtained during the “post” measurement. Only the tests performed under illumination were considered, thanks to the insights gained by observing Figure 5a,b. As the applied potential became more negative, the resistance values decreased exponentially over several orders of magnitude. This behavior has been associated with charge transfer limitations in the reaction mechanisms by Zeng et al. [45] and confirms R3/CPE3 to be the rate-determining component [46]. By focusing on the R3 values at −1.5 V vs. Ag/AgCl (the leftmost value), the catalysts’ values lie in the same order of magnitude (10^1^ Ω). The CP (Figure 5c) sample showed two distinct trends when repeating the measurement. The spread between the “pre” and “post” curves increased as the applied potential decreased, with the two trends becoming similar only at lower potential values. The behavior was interpreted as confirmation that the Cu_2_O catalyst reduced to Cu^0^ during the first part of the measurement, and it became fully reduced when the second part began, which would also explain why the “post” measurements had a more stable and clearer exponential trend. The CS catalysts (Figure 5d) showed almost complete overlap between the “pre” and “post” curves, indicating that the modification of the catalyst during the test was negligible and possibly suppressed by the presence of a thick SnO_2_ outer shell. These observations, along with the lower R3 value, suggested that the stability issues associated with the co-precipitation sample may be improved or resolved by changing the catalyst’s design and synthetic approach to the core–shell structure.

## 4. Conclusions

This study aimed to address the urgent challenge of mitigating CO_2_ levels in the atmosphere and combating global warming by developing earth-abundant catalysts for selective photo-electrochemical CO_2_ conversion. The focus was on comparing two synthetic strategies, namely co-precipitation and core-shell methods, for preparing a Cu_2_O–SnO_2_ catalyst. From a morphological point of view, the two catalysts deeply differed; the CP catalyst’s structure consisted of densely packed agglomerates representing the Cu_2_O bulk material and smaller surface particles attributed to the tin oxide. On the other hand, the CS catalyst was significantly different, consisting of sharpened nanocubes uniformly covered by a thin SnO_2_ shell. The structure exhibited a higher level of organization. The catalysts’ structures played a fundamental role in the band gap energy’s definition. The CP material presented a band gap similar to that of bulk Cu_2_O (2.50 eV), whereas it was significantly higher in the case of the CS catalyst (3.36 eV), resembling that of SnO_2_. It was demonstrated that the novel synthetic core–shell approach enabled tuning of the metallic precursor ratios in the final catalyst. The results from the PEC CO_2_ reduction tests revealed that the Cu_2_O–SnO_2_ core–shell catalyst exhibited a higher photoactivity (24 µA cm^−2^) compared to the co-precipitation-obtained catalyst (18 µA cm^−2^) at a constant applied potential of −0.25 V vs. Ag/AgCl. Likewise, the CS catalyst evidenced a lower onset potential than the CP material under the same conditions in the presence of CO_2_. Regarding the reaction selectivity, a syngas with a CO:H_2_ ratio of ~1.2 was obtained on the CS-tested electrode at −3 mA cm^−2^, a suitable feedstock for hydroformylation reaction. On the other hand, the obtained syngas ratio (~2) for the CP-tested electrode was higher at the same current density, which is an appropriate feed for further methanol synthesis and Fischer–Tropsch processes. In addition, the latter demonstrated the capability of producing C_2_ products, such as ethanol, ascribed to its higher exposition of Cu at the electrode/electrolyte interface. However, the EIS measurements indicated some stability issues observed during the development of the PEC CO_2_ reduction tests, especially for the CP sample. EIS is considered a feasible tool to provide insights on the “health-state” of the catalysts, resulting in a higher global stability of the core–shell structure, probably due to a more effective protective coverage provided by SnO_2_. The ability to tailor the selectivity depending on the synthesis method offers exciting prospects in CO_2_ conversion.

This work is currently framed in the broad context of exploring novel approaches for incorporating Cu_2_O into alternative architectures and environments. Particularly, it was demonstrated how incorporating Cu_2_O into heterojunctions provides simultaneous control over the utilization of charge carriers. The focus was deeply on the stability of the catalyst, and further efforts are needed to improve the photoactivity of future Cu_2_O-based catalysts. The research topic holds great potential for advancing the rational design of Cu_2_O in the field of solar-driven devices.

## Figures and Tables

**Table 1:** Physico-chemical results for the two Cu_2_–SnO_2_ catalysts.

Sample	ICP-OES			N_2_ Physisorption	
	Sn at.%	Cu:Sn	BET Surface Area (m^2^ g^−1^)	Pore Volume (cm^3^ g^−1^)	Pore Size (nm)
CP_CuSn	32.3	2:1	142	0.12	5.9
CS_CuSn	23.4	3:1	64	0.08	5.9

## Data Availability

Our study did not involve any data in external sources.

## Supplementary Materials

The following supporting information can be downloaded at: https://www.mdpi.com/article/10.3390/ma16134497/s1, Figure S1: XRD pattern of the bare CP_Cu_2_O catalyst; Table S1: Molarity and volumes of the reagents involved in the Cu_2_O nanocube synthesis; Figure S2: Reported yield percentages for the Cu_2_O-produced nanoparticles; Figure S3: Characterization of Cu_2_O nanocubes; in particular: (a) XRD pattern, (b,c) SEM images at two different magnifications, and (c) Tauc Plot for the Cu_2_O nanocube band gap energy determination; Figure S4: Blank PEC test of a carbon paper electrode; (a) Linear Sweep Voltammetry performed both in N_2_ and CO_2_ environments with alternating light (2 s); (b) Chronoamperometry test performed at −250 mV, with alternating light every 1 min (d). Faradaic efficiency values of gas and liquid products detected after 2 h of chronopotentiometry in 0.1M KHCO_3_ aqueous electrolyte in two different environments (N_2_ on the left and CO_2_ on the right part of the graph) at applied current density value of −3 mA cm^−2^. All the tests have been conducted with simulated sunlight illumination. Figure S5: Schematic set-up for the development of photo-electrocatalytic tests; Table S2: Relative error of the measurement of Detection Limit concentration for the CO_2_ reduction reaction products; Figure S6: BET isotherms of (a) CP_CuSn catalyst and (b) CS_CuSn catalyst; Figure S7: Comparison of co-precipitation and core–shell Cu_2_O–SnO_2_ (dark and light green lines, respectively) with Cu_2_O samples obtained via co-precipitation (light blue line) and core–shell synthesis (blue line), in particular: (a) LSV curves of the CP samples, (b) LSV curves of the CS samples, and (c) CA curves of all 4 samples; Figure S8: Photo-electrocatalytic tests carried out in N_2_-saturated environment (blue lines) and CO_2_-saturated environment (red lines). In particular: (a) LSV curves of the CP sample both in N_2_ and CO_2_ and (b) LSV curves of CS sample both in N_2_ and CO_2_ environments; Table S3: Productivity values of the two Cu–Sn-based catalysts during 2 h of chronopotentiometry with J_applied_ = −3 mA cm^−2^; Figure S9: Nyquist plots related to (a) CP_CuSn and (b) CS_CuSn samples measured at different potentials vs. Ag/AgCl in CO_2_-saturated electrolyte in tests illuminated by the solar radiation (the points are experimental data). Refs. [26,27,47,48,49,50,51,52,53,54] are cited in the supplementary materials.
