# Supplementary material for: Cu2O/SnO2 Heterostructures: Role of the Synthesis Procedure on PEC CO2 Conversion
Source: Materials (Basel). 2023 Jun 21;16(13):4497. doi: 10.3390/ma16134497 (PMC10342712; doi:10.3390/ma16134497)
Supplement: Supplementary file 1 [file materials-16-04497-s001.zip › materials-2432080-supplementary.pdf]

# $Cu_2O/SnO_2$ Heterostructures: Role of the Synthesis Procedure on PEC $CO_2$ Conversion

**Maddalena Zoli [1], Hilmar Guzmán [1], Adriano Sacco [2], Nunzio Russo [1] and Simelys Hernández [1,*]**

[1] CREST Group, Department of Applied Science and Technology (DISAT), Politecnico di Torino, 10129 Turin, Italy
[2] Center for Sustainable Future Technologies @Polito, Istituto Italiano di Tecnologia, 10144 Turin, Italy
* Correspondence: simelys.hernandez@polito.it

# Supporting Information

S1. Bare $Cu_2O$ synthesis methods ........ 2
S1.1 Co-precipitation ........ 2
S1.2 Core–shell ........ 2
S1.2.1 Preliminary study on the $Cu_2O$ nanoparticles ........ 2
S1.2.2 Details on bare $Cu_2O$ nanocube characterization ........ 4
S2. PEC test onto the Bare Carbon Paper ........ 6
S3. Photo-electrocatalytic setup ........ 7
S4. Additional catalyst characterization results ........ 9
S5. Additional PEC $CO_2$RR results ........ 10
S5.1 Bare $Cu_2O$ PEC tests ........ 10
S5.2 Additional $Cu_2O$–$SnO_2$ PEC results ........ 11
S6. Additional EIS results ........ 13
S7. References ........ 15

# S1. Bare $Cu_2O$ synthesis methods

This section delves into additional details concerning the synthesis and characterization of bare $Cu_2O$ catalysts using the two investigated methods discussed in the main paper. For the catalyst obtained through co-precipitation, designated as CP_$Cu_2O$, the synthesis process is described, and the diffractometric analysis of the material is presented. Regarding the catalyst obtained through core–shell synthesis, while a brief overview of the synthesis is provided, more comprehensive information is offered on preliminary studies conducted prior to the synthesis, as well as the characterization of $Cu_2O$ nanocube particles.

## S1.1 Co-precipitation

A catalyst consisting of pure $Cu_2O$ without any additional additives, referred to as "bare CP_$Cu_2O$," was synthesized and investigated for its potential as a photo-electrocatalyst in the $CO_2$ reduction reaction The results of the test have already been shown by Zoli and co-workers in their first work on a catalyst obtained through co-precipitation in the Supporting Information of their manuscript [1]. The synthesis procedure followed a method similar to the one described in the main text, Section 2.2.1, except that the Sn precursor was not included in the precursor solution. In order to evaluate the effectiveness of the synthesis, the resulting catalyst was subjected to physico-chemical characterization through X-ray diffraction (XRD) analysis of the powdered form. The XRD diffractogram corresponding to this sample can be found in **Figure S1**.

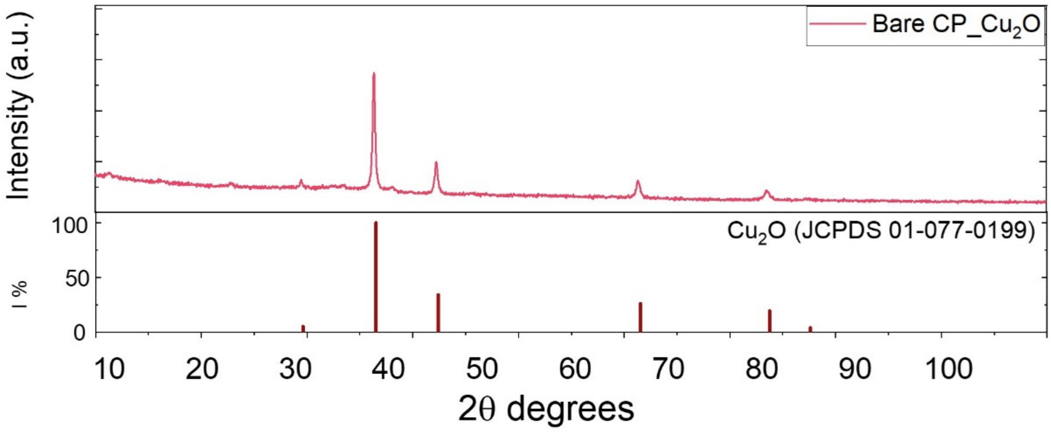


**Figure S1.** XRD pattern of the bare CP_$Cu_2O$ catalyst.

The cubic crystalline phase (cuprite) of $Cu_2O$ (JCPDS 01-077-0199) is the only evident pattern that can be recognized. The most intense peaks corresponded to (111), (200) and (220) orientations at angles of about 36.5°, 42.5°, and 52.6° respectively. No peaks related to CuO or metallic Cu were observed in the diffraction pattern.

## S1.2 Core–shell

### S1.2.1 Preliminary study on the $Cu_2O$ nanoparticles

Starting from the method reported by Zhang *et al.* for the synthesis of $Cu_2O$ nanocubes [2], a first attempt was made to synthesize copper oxide by varying the concentrations and volumes of the reagents to increase the mass of obtained $Cu_2O$. The samples obtained were named as follows: $Cu_2O$-*x*-*y*-*z*-*w*, where *x* is the molarity of $CuCl_2$ (the copper precursor), *y* is the molarity of NaOH (the precipitating agent), *z* is the molarity of $C_6H_8O_6$ (the reductant agent) and *w* is the total volume of the reagent solution. In particular, in $Cu_2O$-0.01-2-0.6-300, nothing changed with respect to the method reported in the literature. In $Cu_2O$-0.01-3-0.6-300, the molarity of the precipitating agent was increased by 50%. $Cu_2O$-0.01-2-0.9-300 had a 50% increase in the molarity of the reducing agent. On the other hand, in $Cu_2O$-0.01-3-0.9-300, both the precipitating and reducing agents increased by 50%. These samples were synthesized with volumes of the solutions equal to those of the starting

synthesis. Then, the changes refer to the volume of the reagents. $Cu_2O$-0.01-3-0.9-600 and $Cu_2O$-0.02-6-1.8-600 were synthesized with doubled volumes, the former maintaining the initial molarities, while the latter's values were doubled. **Table S1** summarizes the molarities and volumes of the solutions used for the syntheses.

**Table S1**. Molarity and volumes of the reagents involved in the $Cu_2O$ nanocube synthesis.

| **Sample** | **Molarity and Volume** | **$CuCl_2$** | **NaOH** | **$C_6H_8O_6$** |
|---|---|---|---|---|
| $Cu_2O$-0.01-2-0.6-300 | M | 0.01 | 2 | 0.6 |
| | mL | 250 | 25 | 25 |
| $Cu_2O$-0.01-3-0.6-300 | M | 0.01 | 3 | 0.6 |
| | mL | 250 | 25 | 25 |
| $Cu_2O$-0.01-2-0.9-300 | M | 0.01 | 2 | 0.9 |
| | mL | 250 | 25 | 25 |
| $Cu_2O$-0.01-3-0.9-300 | M | 0.01 | 3 | 0.9 |
| | mL | 250 | 25 | 25 |
| $Cu_2O$-0.01-3-0.9-600 | M | 0.01 | 3 | 0.9 |
| | mL | 500 | 50 | 50 |
| $Cu_2O$-0.02-6-1.8-600 | M | 0.02 | 6 | 1.8 |
| | mL | 500 | 50 | 50 |

The amounts of copper precursor and $Cu_2O$ obtained from each synthesis were used to calculate the yield percentages. The yields were evaluated in terms of the ratio between the amount of copper in the precursor and amount of copper in the $Cu_2O$ particles obtained. The (S1) was used to calculate the yield:

(S1) $$\eta = \frac{\mathrm{Cu_2O\ amount\ (g)}}{\frac{\mathrm{CuCl_2\ amount\ (g)}}{\mathrm{CuCl_2 molecular\ weight\ (g/mol)}} \cdot \frac{1}{2} \cdot \mathrm{Cu_2O\ molecular\ weight\ (g/mol)}}$$

The resulting yields are reported in **Figure S2**. Following the best result, closest to 100%, the decision was made to implement the doubled concentration values and volumes to carry out the final synthesis for the creation of $Cu_2O$ nanocubes.

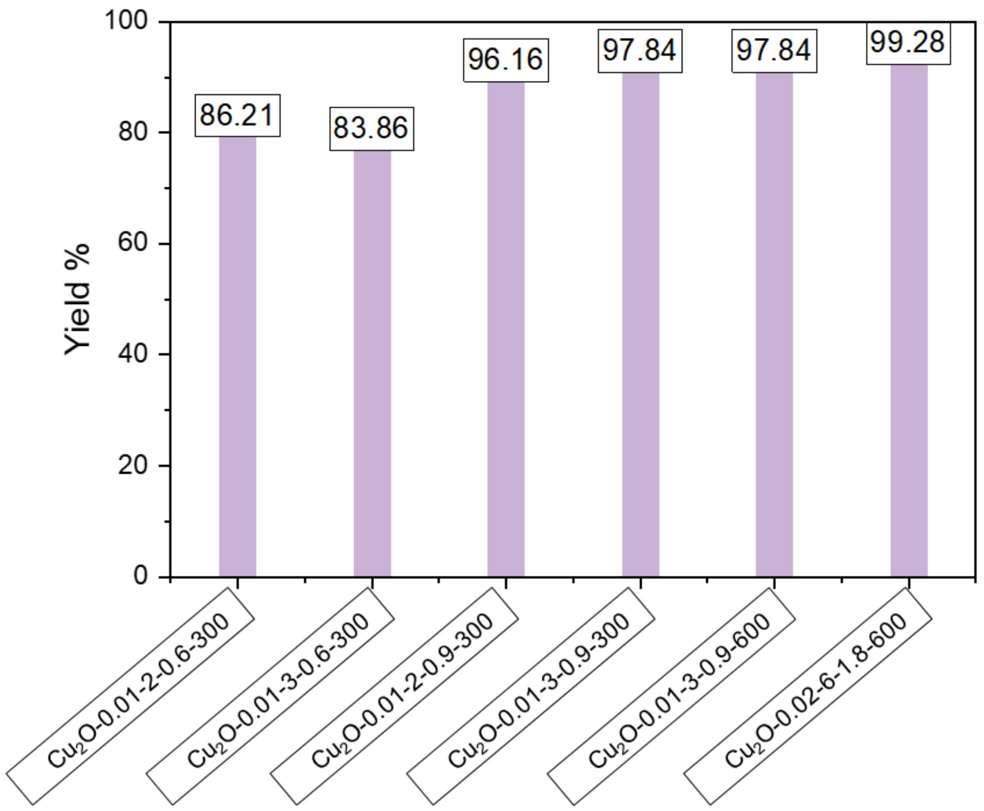


**Figure S2**. Reported yield percentages for the $Cu_2O$-produced nanoparticles.

### S1.2.2 Details on bare $Cu_2O$ nanocube characterization

The as-synthesized $Cu_2O$ nanocubes were characterized to determine their crystal phase. X-ray diffraction analysis was performed, and the results are depicted in **Figure S3**a. The diffractogram exhibited a precise match with the $Cu_2O$ pattern (JCPDS 01-077-0199). The most prominent peaks were observed at approximately 36.5°, 42.5°, and 52.6°, corresponding to (111), (200), and (220) orientations, respectively. Despite the sample's polycrystalline nature, an assessment of the (111) peak intensity relative to the (200) peak intensity was conducted, yielding a value of 3.35. This value suggests the preferential growth of crystals in the (111) orientation. No traces of peaks associated with other copper phases were observed, indicating that the synthesis method employed is highly effective in achieving the desired crystalline phase of $Cu_2O$. The morphology of the nanocubes was examined using a FEI Inspect SEM, and the corresponding micrographs can be found in **Figure S3**b and **Figure S3**c. Both images revealed the smooth surface of the nanocubes, consistent with the findings reported by Zhang *et al.* [2]. The dimensions of the cubes varied (Figure 5.1c), ranging from several hundred nanometers to less than 100 nm. Additionally, energy-dispersive X-ray spectroscopy (EDX) was conducted on different regions of the sample. As anticipated, only two elements were detected: Cu, accounting for an atomic percentage of 66 ± 2; and O, which constituted approximately 33 ± 2% of the sample. Finally, the optical band gap of the bare sample was determined using Tauc's method, which involved analyzing the F(R) spectra acquired from the spectrophotometer in diffuse reflectance mode with an integrating sphere. The energy band gap value obtained, as depicted in **Figure S3**d, was found to be $E_g = 2.43$ eV. This value is slightly higher than the value found in the literature of approximately 2.17 eV [3,4]. However, this disparity could be attributed to the nanometric size of the particles. According to Huang [5], material properties undergo changes based on size, resulting in a band gap value of approximately 2.2 eV for $Cu_2O$ in bulk form and around 2.5 eV for the cubic nanocubes.

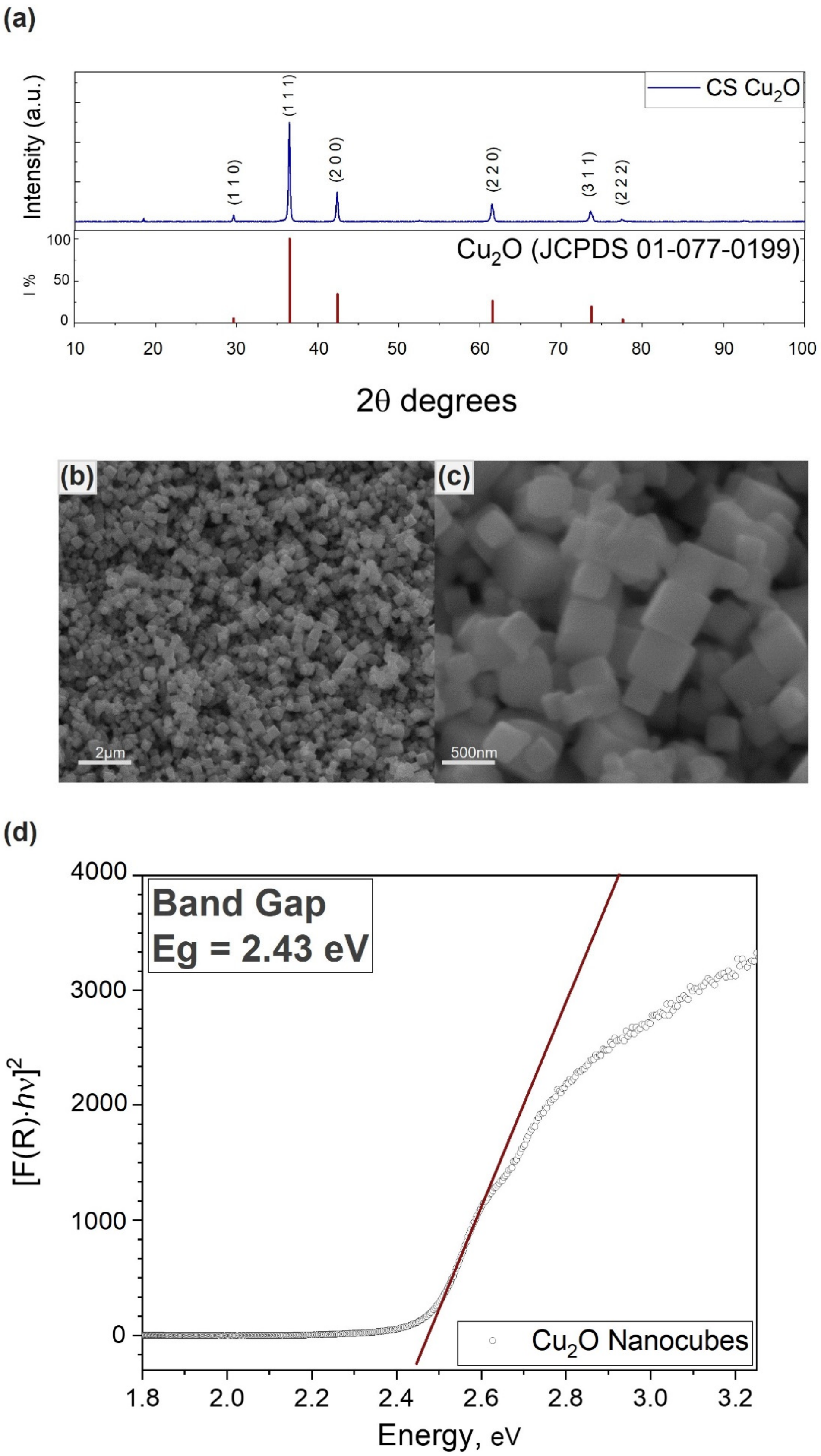


**Figure S3**. Characterization of $Cu_2O$ nanocubes; in particular: (a) XRD pattern, (b) (c) SEM images at two different magnifications, and (c) Tauc Plot for the $Cu_2O$ nanocube band gap energy determination.

# S2. PEC test onto the Bare Carbon Paper

In order to assess the impact of the carbon paper substrate on the photo-electrocatalytic results, a series of blank tests were conducted. These tests involved using the bare carbon paper as the working electrode under different conditions, such as $N_2$- and $CO_2$-saturated electrolytes. The bare electrode was tested with a defined active area of 1cm$^2$ using the experimental set-up and procedure described in Section 2.5 in the main text. This included simulated solar illumination and $N_2$/$CO_2$ bubbling in the aqueous electrolyte. The obtained results from these blank tests are presented in **Figure S4**. By conducting these tests, the aim is to evaluate the specific influence of the carbon paper substrate and ensure the accurate assessment of the catalysts' performance.

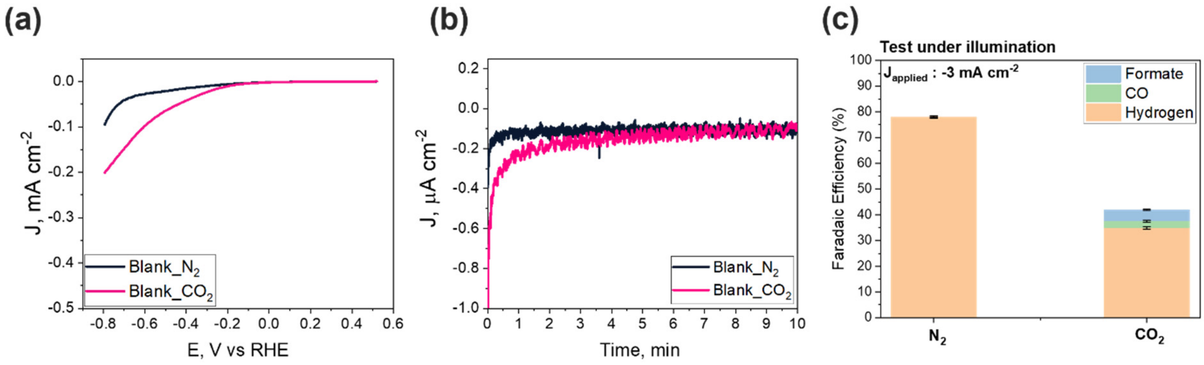


**Figure S4.** Blank PEC test of a carbon paper electrode; (a) Linear Sweep Voltammetry performed both in $N_2$ and $CO_2$ environment with alternating light (2sec); (b) Chronoamperometry test performed at –250 mV, with alternating light every 1 minute (d). Faradaic efficiency values of gas and liquid products detected after 2 hours of chronopotentiometry in 0.1M $KHCO_3$ aqueous electrolyte in two different environments ($N_2$ on the left and $CO_2$ on the right part of the graph) at applied current density value of –3 mA cm$^{-2}$. All the tests have been conducted with simulated sunlight illumination.

The LSV curves (**Figure S4**a) exhibit a nearly zero current density for the test conducted in $N_2$ and a significantly lower activity than the catalytic electrodes for the $CO_2$ bubbling test. The latter shows activity levels approximately one order of magnitude lower compared to the Cu–Sn-based catalysts supported on the carbon paper. As expected, there are no discernible signs of photoactivity in either the LSV or chronoamperometry curves, even when the electrode is exposed to alternating light. The CAs (**Figure S4**b) were carried out at a fixed potential of **–**250 mV, allowing for a comparison between the experiments with the two different $Cu_2O$–$SnO_2$ catalysts. The two curves show no significant differences, as the response obtained under $CO_2$ flow overlaps with that recorded under $N_2$, with a maximum current density value that was two orders of magnitude lower than the ones obtained in the tests with the catalysts (**Figure** 4b of the manuscript). To further investigate the reaction products, two-hour chronopotentiometry tests were performed under simulated sunlight illumination in the presence of the different ($N_2$ or $CO_2$) saturated electrolytes. Without the catalyst particles, an almost exclusively hydrogen evolution reaction (HER) should occur. **Figure S4**c demonstrates that only hydrogen was detected in the $N_2$ environment. A small quantity of $CO_2$ reduction products wwew observed during the test conducted in the $CO_2$ environment at –3 mA cm$^{-2}$ (Faradaic efficiency of 2.5 and 4.5 %, respectively, for CO and formate). In a study conducted by Nursanto et al. [6], comparable results were obtained in blank experiments using bare carbon paper, in which only a minimal production of CO (less than 5%) was observed at -0.8V vs. RHE. Based on the presented tests, it can be concluded that the carbon paper support does not significantly contribute to the formation of $CO_2$ reduction reaction products in the presence of the catalytic layer.

# S3. Photo-electrocatalytic setup

The schematic representation of the set-up discussed in the main article text is presented in **Figure S5**. In the lower part of the figure, the general set-up is depicted, with the double-chamber cell positioned as close as possible to the solar simulator lamp. It is connected to both inlet and outlet gases, as well as to the potentiostat and the μGC. In the inset in the upper part of the figure, a schematic but more detailed representation of the composition of the double-chamber cell is provided, showing each of its two compartments.

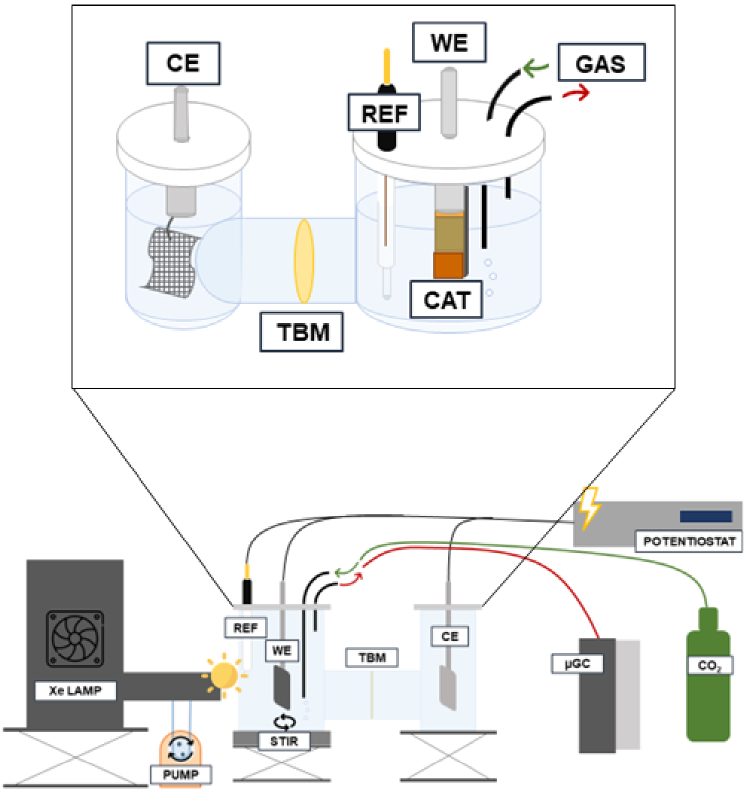


**Figure S5.** Schematic setup for the development of photo-electrocatalytic tests.

To evaluate the photo-electrocatalytic activity of the photocathode, we utilized linear sweep voltammetry (LSV) in both continuous and alternating light modes, as well as chronoamperometry (CA) and chronopotentiometry (CP). The LSV curves were employed to observe how the photocurrent density changes with the applied potential in the system. These curves were repeated multiple times during the test to monitor any variations in photoactivity. On the other hand, the CA curves were used to examine the photocurrent density over time at a fixed potential. We took special precautions when designing the test protocol, particularly in terms of the applied potential range on the working electrode. The aim was to be as conservative as possible to prevent the undesired and uncontrolled reduction of the copper photoactive species and to minimize the coverage of the electrode with Cu(I) oxidation species.

All the instruments used for detection and quantification of $CO_2$ reduction products have been properly calibrated. In **Table S2** we reported for each product of interest its related uncertainty.

**Table S2**. Relative error of the measurement of Detection Limit concentration for the $CO_2$ reduction reaction products.

| **Compound** | **Relative error of Detection Limit concentration measurement (%)** |
|---|---|
| Hydrogen ($H_2$) | 0.47 |
| Carbon Monoxide (CO) | 0.33 |
| Formate ($HCOO^-$) | 0.29 |
| Ethanol ($CH_3CH_2OH$) | 1.05 |

# S4. Additional catalyst characterization results

This section provides additional characterization results of the catalysts, offering further insights into their properties and performance. In particular, the plots of BET isotherms of both analyzed catalysts are reported in **Figure S6**.

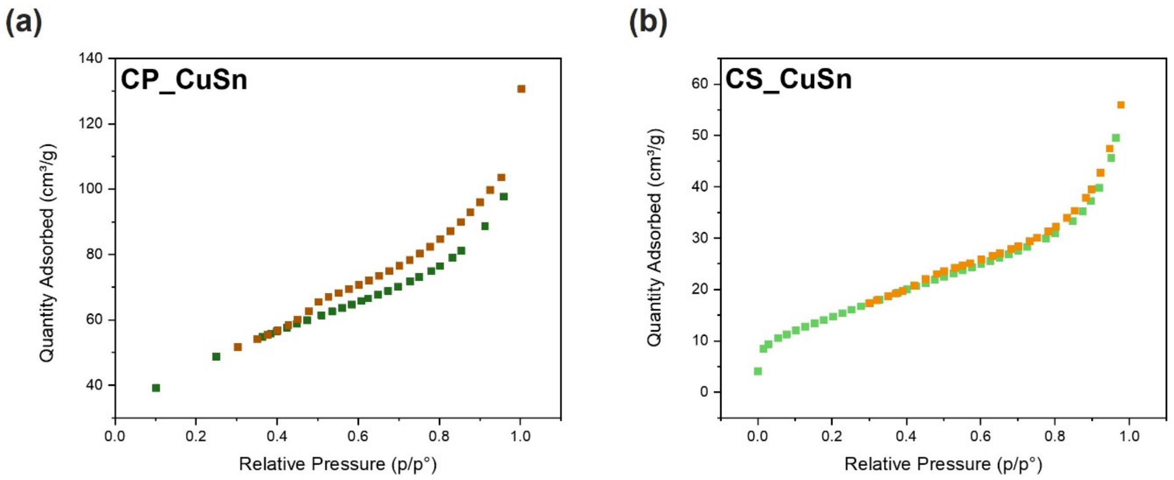


**Figure S6**. BET isotherms of (a) CP_CuSn catalyst, and (b) CS_CuSn catalyst.

A type IV hysteresis, typical of mesoporous materials, can be identified [7]. In fact, the pore size, as reported in Table 1 of the manuscript, falls within the range specified by IUPAC for a material to be considered mesoporous [8]. The higher BET surface area of the CP catalyst can also be correlated with its wider hysteresis, compared to the CS catalyst curve, which, as indicated in Table 1, exhibits a surface area smaller than half of it.

# S5. Additional PEC $CO_2$RR results

## S5.1 Bare $Cu_2O$ PEC tests

In this section, for each Cu–Sn-based catalyst (CP and CS), a comparison with its bare $Cu_2O$ relative sample is provided in terms of LSV and chronoamperometry tests. The plots are shown in **Figure S7**. LSV curves were obtained by conducting the test in a $CO_2$-saturated environment under an alternating simulated solar light, scanning from 0.6 to –0.6 V vs. RHE for the CP samples and from 0.6 to –0.9 V vs. RHE for the CS samples (**Figure S7**a and **Figure S7**b, respectively). The CA tests were conducted for 10 minutes by setting an applied potential of –0.25 V vs. Ag/AgCl. All four samples are reported in the same graph, **Figure S7**c.

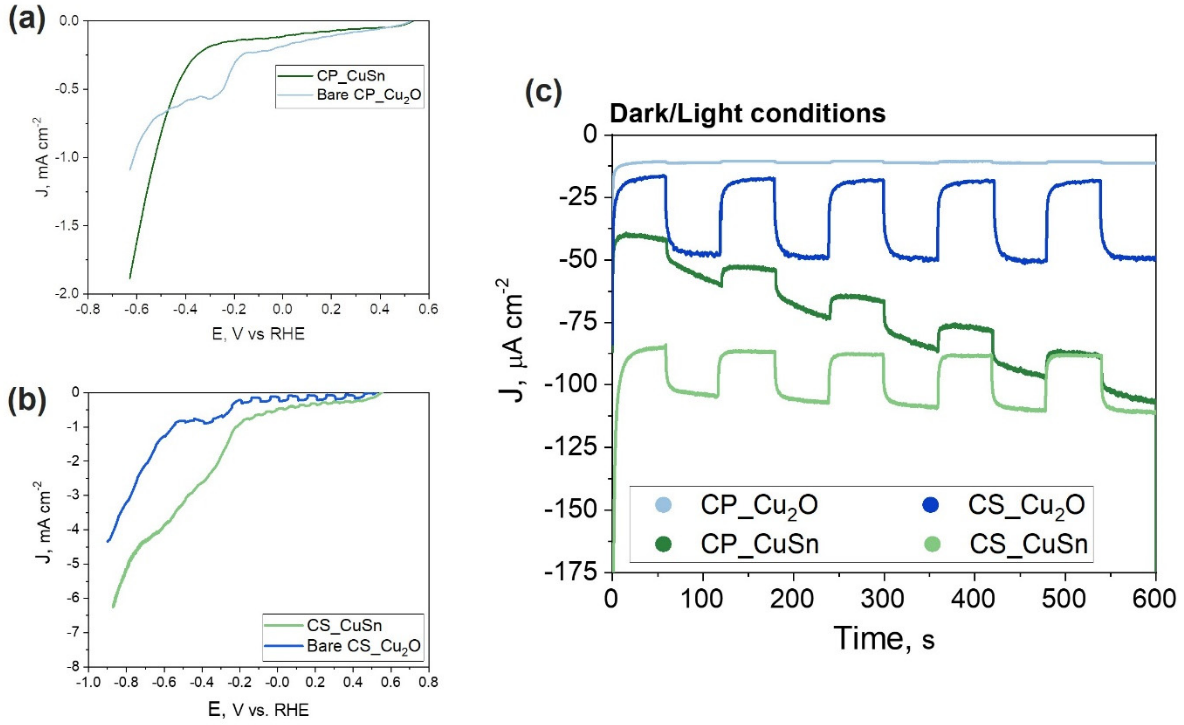


**Figure S7**. Comparison of co-precipitation and core–shell $Cu_2O$–$SnO_2$ (dark and light green lines, respectively) with $Cu_2O$ samples obtained via co-precipitation (light blue line) and core–shell synthesis (blue line), in particular: (a) LSV curves of the CP samples, (b) LSV curves of the CS samples, and (c) CA curves of all 4 samples.

The LSV results of the Cu–Sn based catalysts were already discussed in Section 3.2.1 of the manuscript. It is still possible to observe from the comparison of each $Cu_2O$–$SnO_2$ catalyst with its respective bare $Cu_2O$ that in the tests with the $Cu_2O$ there is a reduction peak present in both cases, positioned between –0.2 and –0.4 V vs. RHE (**Figure S7**a,b). Such a peak could be associated with a reduction of the $Cu^+$ phase of copper due to the reductive testing conditions. When a $SnO_2$ shell of protection for the $Cu^+$ is absent, copper can undergo some phase change due to the testing protocol, leading to a higher overall current density value, as observed by Roy *et al*. [9]. Indeed, the bare CP_$Cu_2O$ (**Figure S7**a) reached a value of about –1 mA $cm^{-2}$ at a potential of –0.6 V vs. RHE, while the CP_CuSn almost reached –2 mA $cm^{-2}$ at the same potential. The same was true for the CS_$Cu_2O$ (**Figure S7**b), which reached a J value of around –4.5 mA $cm^{-2}$ when the potential was –0.9 V vs. RHE, while the CS_CuSn obtained a 40% higher current density value , reaching –6.5 mA $cm^{-2}$ at the same potential. By focusing on the CA tests, shown in **Figure S7**c, the bare CP_$Cu_2O$ (light blue line) revealed a photocurrent gain value of 6 μA $cm^{-2}$, which could be ascribed to the progressive reduction of the $Cu_2O$ species to $Cu^0$, and the consequent loss of photoactive properties. Instead, the $SnO_2$-modified catalyst presented the same photocurrent value obtained in Figure 4c of the manuscript, i.e., 18 μA $cm^{-2}$, and the decreasing trend of its curve (dark green) was in line with the described findings. This result indicated the progressive reduction of the photoactive species during the chronoamperometry test, also confirmed by the enhancement of the dark Faradaic current in comparison to the constant photocurrent gain. Focusing now on the catalysts synthesized

by the core–shell method, both curves were more stable over time, unlike what was observed for the $Cu_2O$–$SnO_2$ CP sample. This could indicate the ability of the core–shell synthesis to provide a more stable and functional $SnO_2$ protection layer compared to the co-precipitation method. The fact that the photocurrent gain observed on bare CS_$Cu_2O$ (blue line) was greater than the one of the $Cu_2O$–$SnO_2$ catalyst (~31 μA cm$^{-2}$ vs. 24 μA cm$^{-2}$) was motivated by the respective band gap (smaller for $Cu_2O$ than $Cu_2O$–$SnO_2$) as observed in the Tauc plots of Figure 3d in the main text and **Figure S3**c in Section S1. In fact, a larger band gap implied that more energy was required to excite an electron from the valance band to the conduction band and, hence, light of a higher frequency and lower wavelength was required [10]. If the band gap is too high, most photons will not cause a photovoltaic effect, causing worse performance in the $CO_2$RR.

## S5.2 Additional $Cu_2O$–$SnO_2$ PEC results

In this section, some insights related to the PEC $CO_2$RR tests of both Cu–Sn-based catalysts are provided. Firstly, for each sample, a comparison between an LSV test performed in an inert atmosphere ($N_2$) and in a $CO_2$-saturated environment is shown (**Figure S***8*). The conditions were the same as those described in Section 3.2.1 of the manuscript.

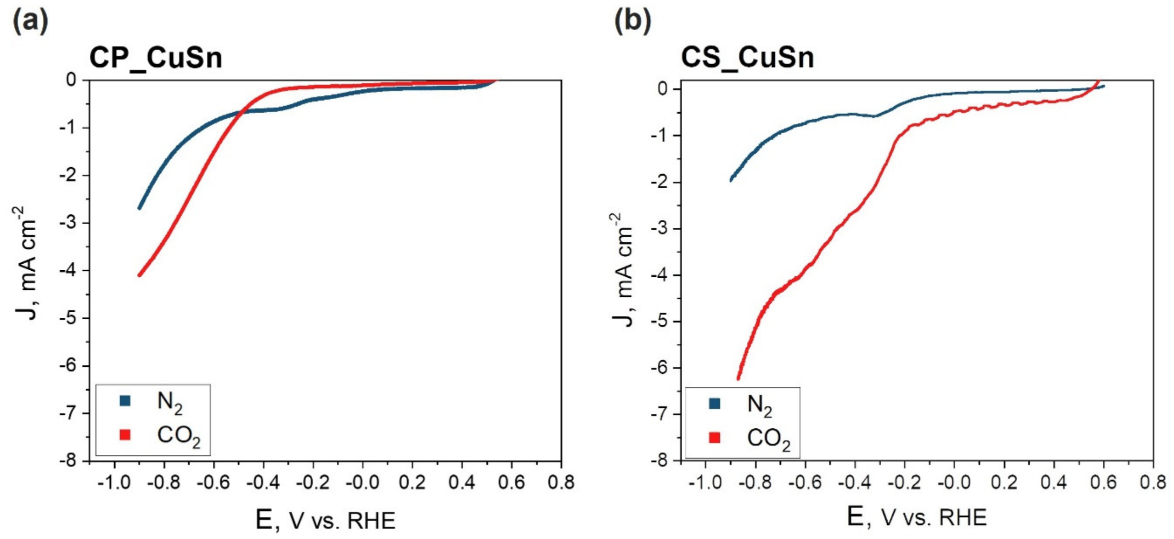


**Figure S8**. Photo-electrocatalytic tests carried out in $N_2$-saturated environment (blue lines) and $CO_2$-saturated environment (red lines). In particular: (a) LSV curves of the CP sample both in $N_2$ and $CO_2$ and (b) LSV curves of CS sample both in $N_2$ and $CO_2$ environments.

The testing protocol was structured to start with the test in an inert atmosphere ($N_2$, blue lines), and to proceed with the $CO_2$-saturated environment test. Therefore, it can be inferred that the peak at –0.4 V vs. RHE in both the blue curves (under $N_2$) may be attributed to the reduction of $Cu^{+1}$ to $Cu^0$. The absence of a similar peak in the tests conducted in a $CO_2$ atmosphere could suggest that the materials had already undergone reduction before being tested under $CO_2$. Furthermore, the discussion regarding the comparison of the maximum values of J could provide some insights. When comparing J values under $CO_2$ and $N_2$, it was observed that they are generally higher with $CO_2$, indicating a greater catalytic activity of the electrode towards $CO_2$ reduction compared to the reduction of $H_2O$ to $H_2$ (water-splitting reaction). Finally, it was worth mentioning that both photo-electrocatalysts exhibited clear evidence of photoactivity, characterized by a wavy pattern resulting from the dark/light alternation every 2 seconds in the first region of the plots. This pattern was more pronounced in the CS catalyst, which can be attributed to its structure, as explained in Section 3.2.1 of the manuscript. Importantly, this photoactivity was observed exclusively within the $CO_2$ atmosphere, while no significant activity was observed in the $N_2$ atmosphere. The absence of activity in the $N_2$ atmosphere implied that the observed photoactivity was specifically associated with the presence of $CO_2$. The contrasting behavior observed between the $CO_2$ and $N_2$ atmospheres may be attributed to the participation of $CO_2$ molecules in the catalytic process, leading to surface reactions and subsequent photo-electrochemical transformations. This

observation was consistent with the intended application of the catalyst for $CO_2$ reduction, where the conversion of $CO_2$ into value-added products was of great significance.

The productivity of the catalysts was determined after a 2-hour chronopotentiometry experiment was also evaluated. The productivity was calculated using the formula:

(2) $$P = (n) / (m_{CAT} \times t).$$

Where n represents the amount of product in millimoles (mmol), $m_{CAT}$ denotes the weight of the catalyst used in the reaction, expressed in grams, and t indicates the duration of the reaction in hours.
This analysis provides valuable insights into the catalysts' performance and their ability to sustain stable and efficient catalytic activity over an extended period of time. The results are expressed in mmol/g·h and they are reported in **Table S3**.

**Table S3.** Productivity values of the two Cu–Sn-based catalysts during 2 hours of chronopotentiometry with $J_{applied}$ = –3 mA $cm^{-2}$.

| | **CP_CuSn** | **CS_CuSn** |
|---|---|---|
| | **(mmol/g·h)** | |
| **$H_2$** | 1.16E-05 | 1.76E-05 |
| **CO** | 1.99E-05 | 2.12E-05 |
| **Formate** | 1.07E-05 | 1.47E-05 |
| **Ethanol** | 8.65E-07 | - |

# S6. Additional EIS results

**Figure S9** presents the Nyquist plots of the two CuSn samples (CP in the higher and CS in the lower part of the figure, respectively). The plots were obtained by measuring the samples at different potentials in the $CO_2$-saturated electrolyte during simulated solar illumination. The insets in figures show the pattern recorded at the lowest applied potential, enlarged to be better understood. As the applied potential became more negative, the impedance magnitude decreased, indicating higher Faradaic reaction rates on the electrode surface. The behavior was the same for both catalysts.

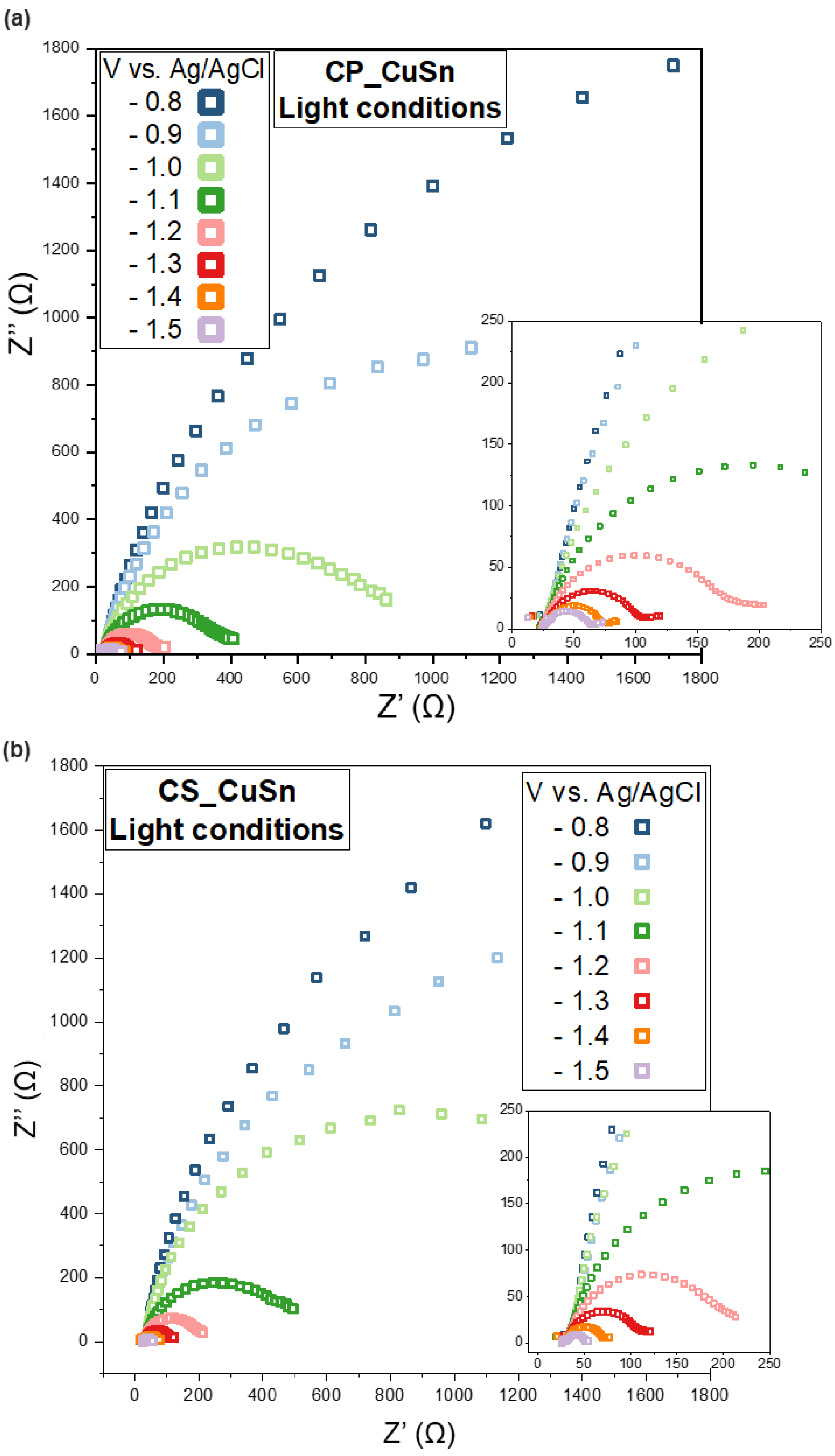


**Figure S9**. Nyquist plots related to (a) CP_CuSn and (b) CS_CuSn samples measured at different potentials vs. Ag/AgCl in $CO_2$-saturated electrolyte in tests illuminated by the solar radiation (the points are experimental data).

# S7. References


1. Zoli, M.; Roldán, D.; Guzmán, H.; Castellino, M.; Chiodoni, A.; Bejtka, K.; Russo, N.; Hernández, S. Facile and Scalable Synthesis of $Cu_2O$-$SnO_2$ Catalyst for the Photoelectrochemical $CO_2$ Conversion. *Catal. Today* **2023**, *415*, doi:10.1016/j.cattod.2022.12.016.
2. Zhang, S.N.; Li, M.; Hua, B.; Duan, N.; Ding, S.; Bergens, S.; Shankar, K.; Luo, J.L. A Rational Design of $Cu_2O$–$SnO_2$ Core-Shell Catalyst for Highly Selective $CO_2$-to-CO Conversion. *ChemCatChem* **2019**, *11*, 4147–4153, doi:10.1002/cctc.201900395.
3. Sawicka-Chudy, P.; Sibiński, M.; Wisz, G.; Rybak-Wilusz, E.; Cholewa, M. Numerical Analysis and Optimization of $Cu_2O$/$TiO_2$, CuO/$TiO_2$, Heterojunction Solar Cells Using SCAPS. *J. Phys. Conf. Ser.* **2018**, *1033*, 0–10, doi:10.1088/1742-6596/1033/1/012002.
4. Ravichandran, A.T.; Dhanabalan, K.; Vasuhi, A.; Chandramohan, R.; Mantha, S. Morphology, Bandgap, and Grain Size Tailoring in $Cu_2O$ Thin Film by SILAR Method. *IEEE Trans. Nanotechnol.* **2015**, *14*, 108–112, doi:10.1109/TNANO.2014.2369438.
5. Huang, M.H. Semiconductor Nanocrystals Possessing Broadly Size- and Facet-Dependent Optical Properties. *J. Chinese Chem. Soc.* **2021**, *68*, 45–50, doi:10.1002/jccs.202000267.
6. Nursanto, E.B.; Jeon, H.S.; Kim, C.; Jee, M.S.; Koh, J.H.; Hwang, Y.J.; Min, B.K. Gold Catalyst Reactivity for $CO_2$ Electro-Reduction: From Nano Particle to Layer. *Catal. Today* **2016**, *260*, 107–111, doi:10.1016/j.cattod.2015.05.017.
7. Thommes, M.; Kaneko, K.; Neimark, A. V.; Olivier, J.P.; Rodriguez-Reinoso, F.; Rouquerol, J.; Sing, K.S.W. Physisorption of Gases, with Special Reference to the Evaluation of Surface Area and Pore Size Distribution (IUPAC Technical Report). *Pure Appl. Chem.* **2015**, *87*, 1051–1069, doi:10.1515/pac-2014-1117.
8. Kaneko, K. Determination of Pore Size and Pore Size Distribution. 1. Adsorbents and Catalysts. *J. Memb. Sci.* **1994**, *96*, 59–89, doi:10.1016/0376-7388(94)00126-X.
9. Roy, A.; Jadhav, H.S.; Gil Seo, J. $Cu_2O$/CuO Electrocatalyst for Electrochemical Reduction of Carbon Dioxide to Methanol. *Electroanalysis* **2021**, *33*, 705–712, doi:10.1002/elan.202060265.
10. Cox, P.A. *The Electronic Structure and Chemistry of Solids*; Oxford Uni.; Oxford [Oxfordshire], 1987; ISBN 9780198552048.
